# Camptothecin transformation in plant-associated Sphingobium lineages is linked to a plasmid accessory region

**DOI:** 10.1016/j.crmicr.2026.100677

**Published:** 2026-10-23

**Authors:** Hisashi Takeda, Nagomi Takahashi, Kyle Shuta Ikeda Kakiura, Hitomi Suzuki, Tatsuki Kijinami, Masako Horiuchi, Daigo Wakana, Hiroshi Sudoh, Tomoo Hosoe

**Affiliations:** a Laboratory of Bioregulatory Science, Hoshi University, 2-4-41 Ebara, Shinagawa, Tokyo, 142-8501, Japan; b Laboratory of Botany, Hoshi University, 2-4-41 Ebara, Shinagawa, Tokyo, 142-8501, Japan

**Keywords:** Camptothecin, *Sphingobium*, Alkaloid transformation, Rhizosphere, Accessory genome, Comparative genomics, Oxygenase

## Abstract

Specialized plant metabolites impose strong chemical constraints on plant-associated microorganisms; however, the bacterial pathways that enable transformation of complex alkaloid scaffolds remain poorly understood. Here, we show that plant-associated *Sphingobium* lineages transform camptothecin (CPT) and that this phenotype is associated with a plasmid accessory region. Strain OP1, isolated from the rhizosphere of the CPT-producing plant *Ophiorrhiza pumila*, transformed CPT into a transient product, M1, which retained the E-ring lactone and subsequently decreased during incubation. OP1 also transformed 10-hydroxycamptothecin, whereas transformation of 7-ethyl-10-hydroxycamptothecin was delayed or limited, indicating substrate selectivity. Genome-based taxonomic analyses placed OP1, NN19, and KJ09 in one putative novel *Sphingobium* species-level lineage and IROP2 in a related but distinct lineage. Comparison of NN19 with its non-transforming derivative NTG18 resulted in the identification of GAP2, a 97.8-kb region of plasmid pNN19-1 absent from NTG18 and enriched in redox, hydrolase, transport, regulatory, oxygenation, and ring-cleavage functions. A rearranged pIROP2 module retained homologs of the core GAP2 oxygenation and ring-cleavage candidates, whereas IROP2 generated oxygenated products from CPT analogs. RNA sequencing during growth of NN19 with CPT as the sole added carbon source prioritized the same early module, including monooxygenase and dioxygenase candidates. The collective phenotypic, chemical, genetic, comparative-genomic, and expression evidence supports a model in which a plasmid accessory module contributes to CPT transformation.

## Highlights

• Plant-associated *Sphingobium* lineages transform camptothecin (CPT).
• OP1 grows with CPT as the sole added carbon source and transiently forms M1.
• Loss of the 97.8-kb plasmid GAP2 region accompanies loss of CPT transformation.
• GAP2 contains an expressed oxygenation/ring-cleavage candidate module.
• This module is conserved in a distinct CPT-transforming *Sphingobium* lineage.

## Graphical abstract

**Figure:**
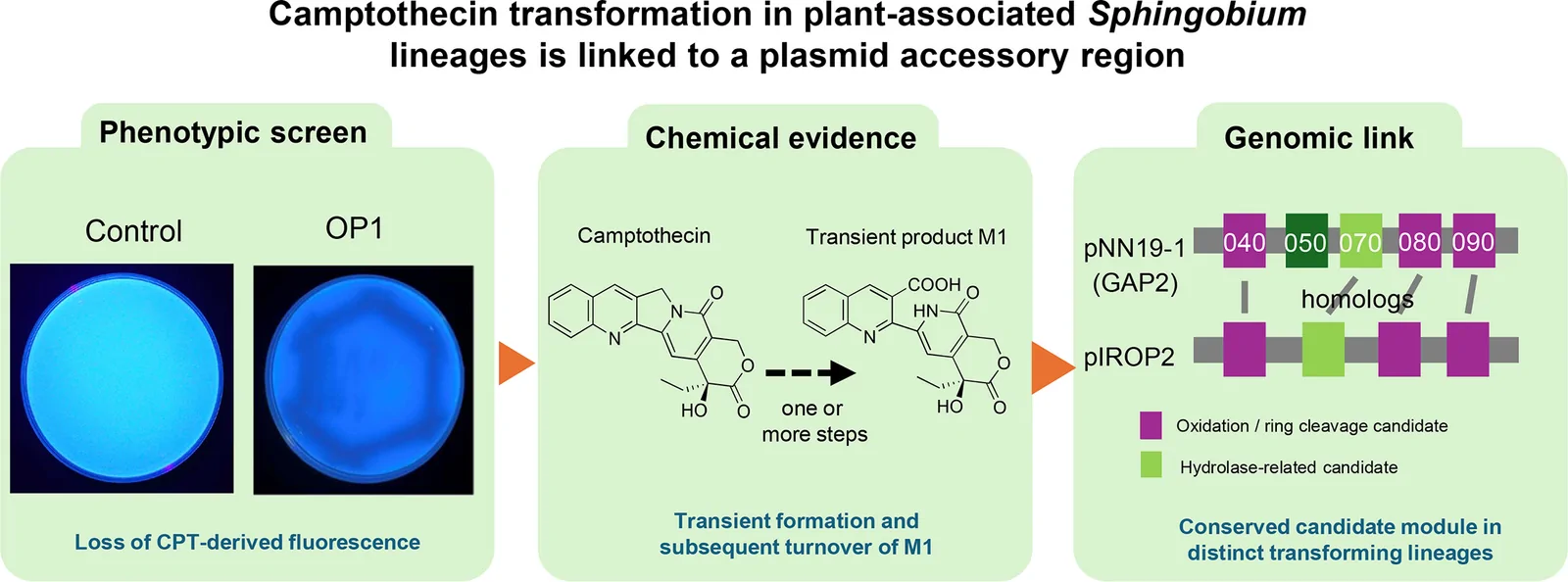

## 1 Introduction

Plant secondary metabolites play a central role as “chemical currencies” in plant-associated microbial habitats. These secondary metabolites can act as defense molecules, allelochemicals, or signals, and their persistence in soil and rhizosphere environments is affected by not only abiotic processes but also microbial transformation and degradation (Jilani et al., 2008; Nakayasu et al., 2023; Pang et al., 2024; Sugiyama, 2023). Microbial metabolism of plant-derived aromatic compounds has been studied extensively, especially with regard to relatively simple phenolic compounds and xenobiotic-like substrates. By contrast, much less is known about how bacteria metabolize structurally complex plant alkaloids (Jilani et al., 2008; Rathbone and Bruce, 2002). This knowledge gap is important in microbial ecology because alkaloids can significantly impact the microbial community structure, and this lack of information is also relevant in biotechnology because microbial enzymes can selectively transform complex natural products under mild conditions (Rathbone and Bruce, 2002).

Camptothecin (CPT) is a pentacyclic quinoline alkaloid and the lead scaffold of clinically used topoisomerase I inhibitors such as irinotecan and topotecan (Hartmann and Lipp, 2006; Hsiang et al., 1985; Mathijssen et al., 2002; Wall et al., 1966). Investigations of CPT have therefore primarily been conducted from pharmacological, biosynthetic, medicinal chemistry, and biotechnological perspectives (El-Hady et al., 2024; Hartmann and Lipp, 2006; Mathijssen et al., 2002; Soujanya et al., 2017). However, CPT is also a specialized metabolite that occurs in root-associated tissues of CPT-producing plants, including *Ophiorrhiza pumila* Champ. ex Benth. (Lee et al., 2020; Sudo et al., 2002; Yamazaki et al., 2007). The release of CPT or CPT-related compounds from such plants can expose rhizosphere microorganisms to a bioactive, nitrogen-containing aromatic scaffold. This ecological setting raises a distinct microbiological question: can plant-associated bacteria transform or use CPT? If so, which organisms, metabolites, and genes mediate the entry of this alkaloid into bacterial metabolic pathways?

Details regarding the bacterial metabolism of CPT remain largely unresolved. Environmental studies have examined CPT degradation in other contexts, including marine antifouling applications, in which photodegradation was found to be rapid, but biodegradation in seawater was reportedly negligible (Hao et al., 2022). The organisms responsible for CPT turnover in plant-associated environments, the transient metabolites formed during bacterial transformation, and the genetic determinants of these reactions have not been clearly identified. From a biochemical perspective, entry into the CPT scaffold likely requires oxygenation of the aromatic or polycyclic core, followed by ring opening and downstream hydrolytic or redox reactions. Such reactions are consistent with enzyme families involved in aromatic compound metabolism, including Rieske/non-heme iron oxygenases, flavin-dependent monooxygenases, extradiol dioxygenases, and hydrolases (Barry and Challis, 2013; Fetzner, 2012; Kweon et al., 2008; Lipscomb, 2008; Pati et al., 2022; Xu et al., 1997). Nevertheless, no bacterial genetic module has been linked to CPT transformation.

Members of the genus *Sphingobium* provide a useful framework for investigating this problem. *Sphingobium* species belong to the sphingomonad group of Alphaproteobacteria and include soil-, water-, and plant-associated bacteria frequently recovered from chemically selective environments. Many of the *Sphingobium* strains characterized thus far degrade aromatic or otherwise recalcitrant organic compounds, and the mediators of these activities are often encoded by accessory replicons or plasmid-associated catabolic regions (Nagata et al., 2006, 2007, 2010; Romine et al., 1999; Stolz, 2014). The well-characterized strain *Sphingobium* sp. SYK-6 further illustrates the capacity of this genus to process diverse lignin-derived monoaryl and biaryl compounds (Masai et al., 2012). Furthermore, *Sphingobium* sp. BD3100 can reportedly utilize the protoberberine alkaloids berberine and palmatine to produce hydroxylated alkaloid derivatives (Takeda et al., 2015). Such mobile or rearrangeable catabolic architectures enable the rapid acquisition and remodeling of metabolic functions, making *Sphingobium* a plausible reservoir of enzymes for processing plant-derived aromatic metabolites. However, CPT-transforming activity is likely not a general property of the genus. Establishing a genetic basis for CPT metabolism will therefore require connecting phenotype and metabolite chemistry with comparative genomic data, loss-of-function evidence, conservation across independent transforming lineages, and gene expression during growth with CPT.

In this study, we isolated CPT-transforming *Sphingobium* strains from plant-associated environments and integrated phenotypic assays, metabolite chemistry, genome-based taxonomy, comparative genomics, cross-lineage conservation, and RNA sequencing to define a candidate genomic framework associated with CPT transformation. Strain OP1, isolated from the rhizosphere of *O. pumila*, transformed CPT into a transient product, M1, which retained the E-ring lactone and subsequently decreased during incubation. OP1 viable counts also increased when CPT was supplied as the sole added carbon source. Genome-based taxonomic analyses placed OP1, NN19, and KJ09 in one putative novel species-level lineage and IROP2 in a related but distinct lineage. Comparison of NN19 with the non-transforming NN19-derived mutant NTG18 identified GAP2, a plasmid-associated region enriched in oxygenation, ring-cleavage, redox, hydrolase, transport, and regulatory candidates. Integrating deletion mapping, cross-lineage conservation, functional annotation, and expression during growth with CPT prioritized a compact oxygenation/ring-cleavage module within GAP2. We propose a testable model in which plasmid-associated accessory metabolism enables plant-associated *Sphingobium* lineages to transform a structurally complex plant alkaloid and may contribute to adaptation in chemically selective plant-associated habitats.

## 2 Results

### 2.1 Isolation and characterization of a CPT-transforming *Sphingobium* strain

To identify microorganisms involved in the environmental transformation of CPT, rhizosphere soil associated with the CPT-producing plant *Ophiorrhiza pumila* was subjected to enrichment culture in an inorganic salts medium containing CPT as the sole added carbon source. After repeated enrichment, colonies were obtained on CPT-containing agar plates. One isolate exhibited a clear local reduction in CPT-derived fluorescence under irradiation with 365-nm ultraviolet light-emitting diode, indicating localized depletion or transformation of CPT around the colony (Fig. 1). The isolate formed wet, yellow colonies on LB agar and was designated strain OP1.

**Fig. 1:**
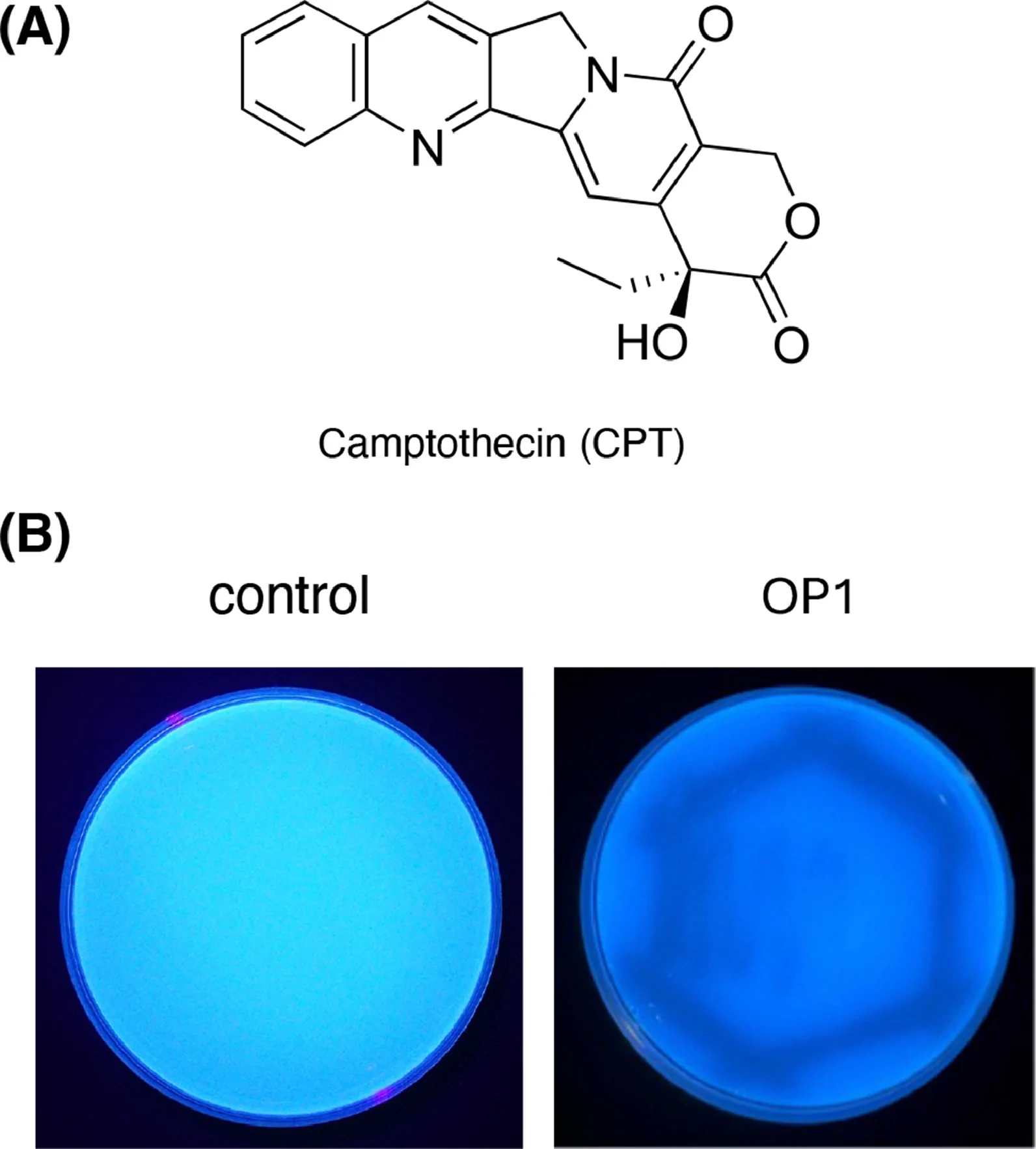
Fluorescence-based screening of CPT transformation by strain OP1. (A) Chemical structure of camptothecin (CPT). (B) CPT-containing agar medium inoculated with strain OP1 or left uninoculated as the cell-free control. Plates were photographed under illumination with a 365-nm ultraviolet light-emitting diode. The localized reduction in CPT-derived fluorescence around OP1 growth was used as a qualitative indicator of CPT depletion or transformation.

Initial taxonomic placement based on the 16S rRNA gene sequence assigned OP1 to the genus *Sphingobium*. Although OP1 was related to described *Sphingobium* species in the 16S rRNA gene tree, it formed a distinct branch from the tested reference strains (Supplementary Figure S1). In contrast to OP1, the reference strains *S. japonicum, S. chlorophenolicum*, and *S. yanoikuyae* showed little change in CPT abundance during incubation in CPT-containing inorganic salts medium (Supplementary Figure S2A). These observations identified OP1 as a CPT-transforming Sphingobium strain.

Because suspended CPT interfered with optical-density measurements, growth of OP1 in inorganic salts medium was evaluated by viable-cell enumeration. In medium containing CPT as the sole added carbon source, the mean viable count increased from 5.3 ± 0.7 log10 CFU/mL at 0 h to 8.6 ± 0.1 log10 CFU/mL at 48 h (n = 3 independent cultures) and subsequently declined while remaining detectable at 120 h (Supplementary Figure S2B). In the CPT-free control, the viable count did not increase, and all three cultures were below the detection limit (<100 CFU/mL) at 96 and 120 h. In parallel, CPT abundance decreased during incubation with OP1 and was below the limit of quantification at 60 and 72 h, whereas it remained near the initial level in cultures of the three reference *Sphingobium* strains (Supplementary Figure S2A). The culture pH remained essentially unchanged, with mean values of 7.31 ± 0.01, 7.33 ± 0.01, and 7.33 ± 0.01 at 0, 48, and 120 h, respectively (mean ± SD, n = 3). Thus, the increase in OP1 viable counts accompanied CPT depletion without a measurable shift in bulk culture pH.

### 2.2 Genome-based taxonomic analysis of CPT-transforming isolates

Because additional CPT-transforming isolates were obtained from different sampling sources and used for comparative genomic analyses, the genome-based taxonomic relationships between these isolates were examined. Whole-genome taxonomic analysis using the Type (Strain) Genome Server (TYGS) placed OP1, NN19, and KJ09 in a single species-level cluster (Fig. 2; Supplementary Tables S1A–S1C) (Meier-Kolthoff and Göker, 2019). Pairwise digital DNA-DNA hybridization (dDDH) values for OP1, NN19, and KJ09 were 100.0% using the recommended GGDC formula *d_4_*, indicating that these three isolates belong to the same genome/species-level group. The genomic features of the genome-sequenced isolates and NN19-derived mutant NTG18 are summarized in Table 1 and Supplementary Table S2. The genome size and G+C content of the isolates were also highly similar, as follows: OP1, 3054,081 bp and 62.0% G+C; KJ09, 3054,235 bp and 62.0% G+C; and NN19, 3060,055 bp and 61.9% G+C.

**Fig. 2:**
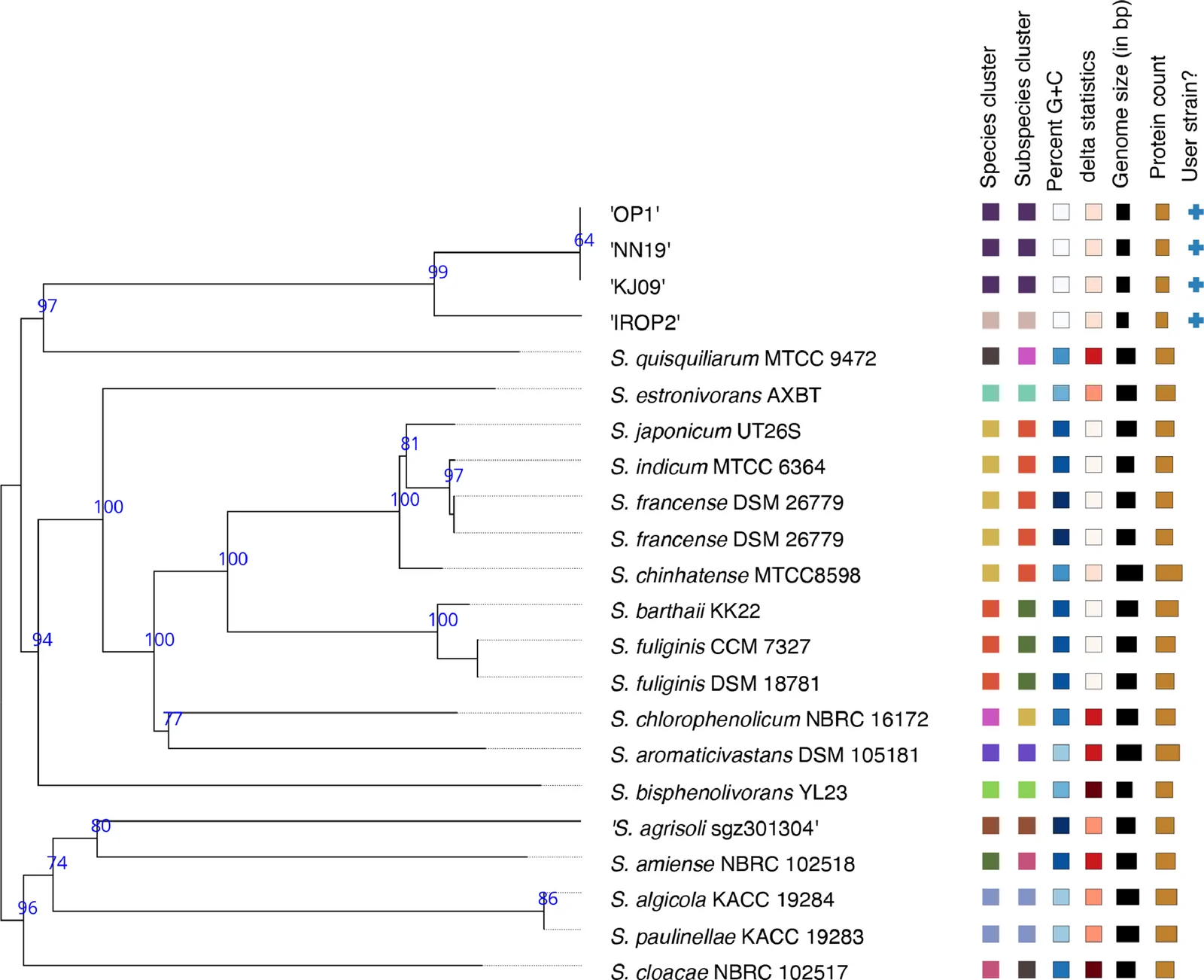
Genome-based taxonomy of CPT-transforming isolates. Genome-scale Genome BLAST Distance Phylogeny (GBDP) tree inferred by the Type (Strain) Genome Server (TYGS) from whole-genome sequences of OP1, NN19, KJ09, IROP2, and related type strains. Branch lengths were scaled using GBDP distance formula *d_5_*, and branch labels indicate GBDP pseudo-bootstrap support values >60% from 100 replications. OP1, NN19, and KJ09 formed a single species-level cluster, whereas IROP2 formed a related but distinct lineage. Metadata columns summarize TYGS species and subspecies clustering, G+C content, genome size, protein count, and user-genome status.

**Table 1:** Isolates used and their roles in the present study.

**Strain**	**Origin**	**CPT phenotype**	**Role in this study**	**Genome-based placement**
NN19	Medicinal plant garden, Hoshi University	CPT-transforming	Genome, NTG18 comparison, and RNA-seq representative of OP1/NN19/KJ09 lineage	OP1/NN19/KJ09 novel species-level cluster
KJ09	Medicinal plant garden, Hoshi University	CPT-transforming	Additional CPT-transforming isolate in OP1/NN19/KJ09 lineage	OP1/NN19/KJ09 novel species-level cluster
OP1	Medicinal plant garden, Hoshi University	CPT-transforming	Phenotype and metabolite-characterization representative of OP1/NN19/KJ09 lineage	OP1/NN19/KJ09 novel species-level cluster
IROP2	Iriomote Island	CPT-transforming	CPT-transforming isolate from a distinct but related species-level lineage	Distinct related novel species-level lineage
NTG18	NN19-derived mutant	Non-transforming	Non-transforming mutant derived from NN19; deletion comparator	Not included in TYGS four-strain classification; NN19-derived mutant

*Abbreviations:* CPT, camptothecin; TYGS, Type (Strain) Genome Server.

By contrast, strain IROP2, which was isolated on Iriomote Island, Japan, formed a sister lineage to the OP1/NN19/KJ09 cluster. Pairwise dDDH values between IROP2 and OP1, NN19, or KJ09 were 57.0% according to formula *d_4_*, below the 70% species boundary. IROP2 also had a smaller assembled genome size of 2858,869 bp and a G+C content of 62.2%. Relative to available type-strain genomes, TYGS identified the OP1/NN19/KJ09 cluster and IROP2 lineage as potentially novel species-level lineages. Thus, the CPT-transforming isolates analyzed here comprise one putative novel *Sphingobium* species-level lineage represented by OP1, NN19, and KJ09, and a closely related but distinct putative novel lineage represented by IROP2.

The results of these taxonomic analyses provided the framework for subsequent experiments. OP1 was used as the principal strain for phenotypic and metabolite characterization, whereas NN19 served as a representative strain for genome and transcriptome analyses within the OP1/KJ09/NN19 lineage, and KJ09 was an additional same-lineage CPT-transforming isolate, with IROP2 as a related but genomically distinct CPT-transforming lineage.

### 2.3 OP1 transforms CPT into a transient product, M1

Whether OP1 actively transforms CPT was evaluated using resting-cell assays in which cells were grown in LB medium, washed, and then incubated with CPT in phosphate buffer. HPLC analysis showed that the level of CPT decreased markedly in the presence of OP1 cells, whereas little or no decrease was observed in cell-free controls or in assays using non-transforming reference *Sphingobium* strains. These results indicated that the disappearance of CPT was dependent on OP1 cells rather than due to spontaneous degradation under the assay conditions used.

Whether prior exposure to CPT is required for CPT-transforming activity was evaluated using resting-cell assays with cells precultured in LB medium with and without CPT. CPT transformation proceeded similarly with both cells exposed to CPT and un-exposed LB-grown cells, indicating that detectable CPT-transforming activity does not require strong prior exposure to CPT under the conditions examined in this study (Fig. 3A).

**Fig. 3:**
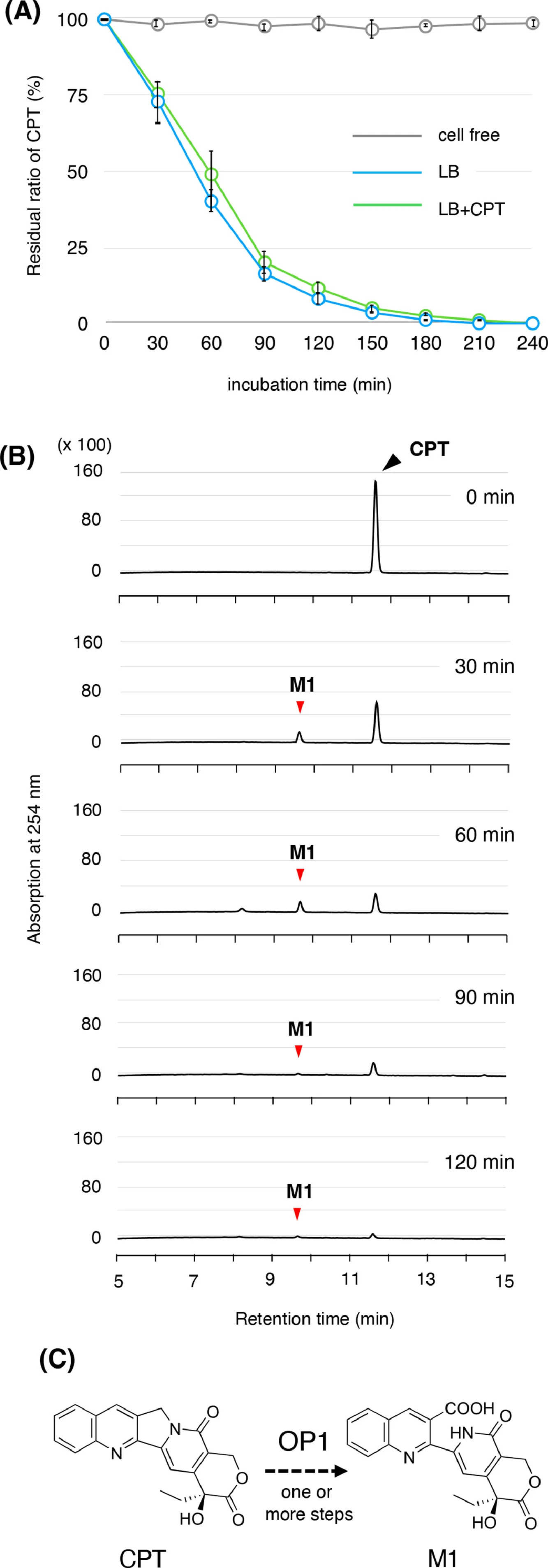
OP1 transforms CPT and produces the transient product M1. (A) Time-dependent decrease in CPT level in resting-cell assays. OP1 resting cells reduced the CPT abundance, whereas cell-free controls and non-transforming reference *Sphingobium* strains showed little or no decrease in CPT under the same conditions. Data points represent the mean of three independent experiments, and error bars indicate the standard deviation (n = 3). (B) High-performance liquid chromatography (HPLC) chromatograms of the OP1 resting-cell reaction analyzed over time. The intensity of the CPT peak decreased, and a metabolite peak designated M1 accumulated transiently before decreasing during prolonged incubation. Arrowheads indicate the CPT and M1 peaks. (C) Structure of purified M1 determined by mass spectrometry and nuclear magnetic resonance analyses.

During incubation of resting OP1 cells, new HPLC peaks appeared concomitantly with the decrease in the CPT peak (Fig. 3B). No M1 peak was detected in the cell-free control. One of the prominent metabolite peaks, designated M1, was purified and subjected to MS and NMR analyses. The molecular formula of M1 was determined as C_20_H_16_N_2_O_6_ by high-resolution electrospray ionization mass spectrometry in positive-ion mode: C_20_H_17_N_2_O_6_ [M+H]^+^ (observed m/z 381.1107; calculated m/z 381.1087; mass difference, 2.00 mmu). One- and two-dimensional nuclear magnetic resonance spectroscopy supported the structure shown in Fig. 3C, and the complete signal assignments are provided in Supplementary Table S3 and Supplementary Figures S3–S5. Notably, M1 retained the E-ring lactone of CPT and was therefore distinct from the carboxylate generated by simple pH-dependent hydrolysis of this ring. In time-course HPLC profiles, M1 transiently accumulated during the early phase of the reaction and subsequently decreased with prolonged incubation. This behavior is consistent with further transformation of M1 rather than accumulation as a stable end product.

### 2.4 Substrate-selective transformation of CPT analogs

Whether the CPT-transforming activity of OP1 is substrate selective was evaluated by testing OP1 against various CPT analogs, including 10-hydroxycamptothecin (HCPT) and 7-ethyl-10-hydroxycamptothecin (EHCPT). OP1 transformed HCPT, as indicated by the loss of substrate-derived fluorescence and detection of new metabolite peaks by HPLC (Supplementary Figures S6A and S6B). These results are consistent with oxygenative transformation of the HCPT scaffold.

In contrast to HCPT, transformation of EHCPT by OP1 was delayed or limited. Although EHCPT levels decreased under some assay conditions and new peaks were detected with longer incubation, both the apparent reaction rate and product-peak profile differed from those of CPT and HCPT (Supplementary Figures S6A and S6C). LC–ESI-TOF-MS analyses revealed two OP1-derived EHCPT products: one consistent with C_22_H_21_N_2_O_6_ [M+H]^+^ (observed *m*/*z* 409.14136; calculated *m*/*z* 409.13996; mass difference, 1.40 mmu) and corresponding to monooxygenation, and another consistent with C_22_H_21_N_2_O_7_ [M+H]^+^ (observed *m*/*z* 425.13287; calculated *m*/*z* 425.13488; mass difference, −2.00 mmu) and corresponding to dioxygenation. These results indicate that OP1-mediated transformation is sensitive to structural differences among CPT analogs. The distinct behaviors of HCPT and EHCPT are consistent with an enzyme-mediated process that recognizes specific features of the CPT scaffold and in which oxygenation of the aromatic/polycyclic core is an early transformation step.

### 2.5 Definition of the GAP2 candidate region by comparison with NTG18

The OP1 metabolite data demonstrated chemical transformation of CPT, but the responsible genes were unknown. The genomic regions associated with CPT transformation were identified by comparing NN19 with NTG18, a non-transforming mutant derived from NN19. Strain NTG18 was selected as a mutant that failed to grow on CPT-containing inorganic salts medium. The non-transforming phenotype of NTG18 was further confirmed by the absence of CPT-derived fluorescence loss on CPT-containing R2A plates and by HPLC-based resting-cell assays showing no detectable CPT transformation. The growth rate of NTG18 was comparable to that of wild-type NN19 under nonselective conditions, indicating that the phenotype could not be explained by a general growth defect. Consistent with this phenotypic observation, a whole-genome comparison did not provide evidence suggesting a major loss of core chromosomal regions or broadly conserved growth-related functions. Whole-genome alignment analyses showed that NTG18 aligned almost completely with NN19 at the chromosomal level but lacked several NN19 plasmid regions (Supplementary Table S4A). The most prominent losses were located on pNN19-1 and included a 10,083-bp region (R2; pNN19-1 positions 401,277 to 411,359) and an 86,997-bp region (R3; pNN19-1 positions 411,463 to 498,459) (Fig. 4).

**Fig. 4:**
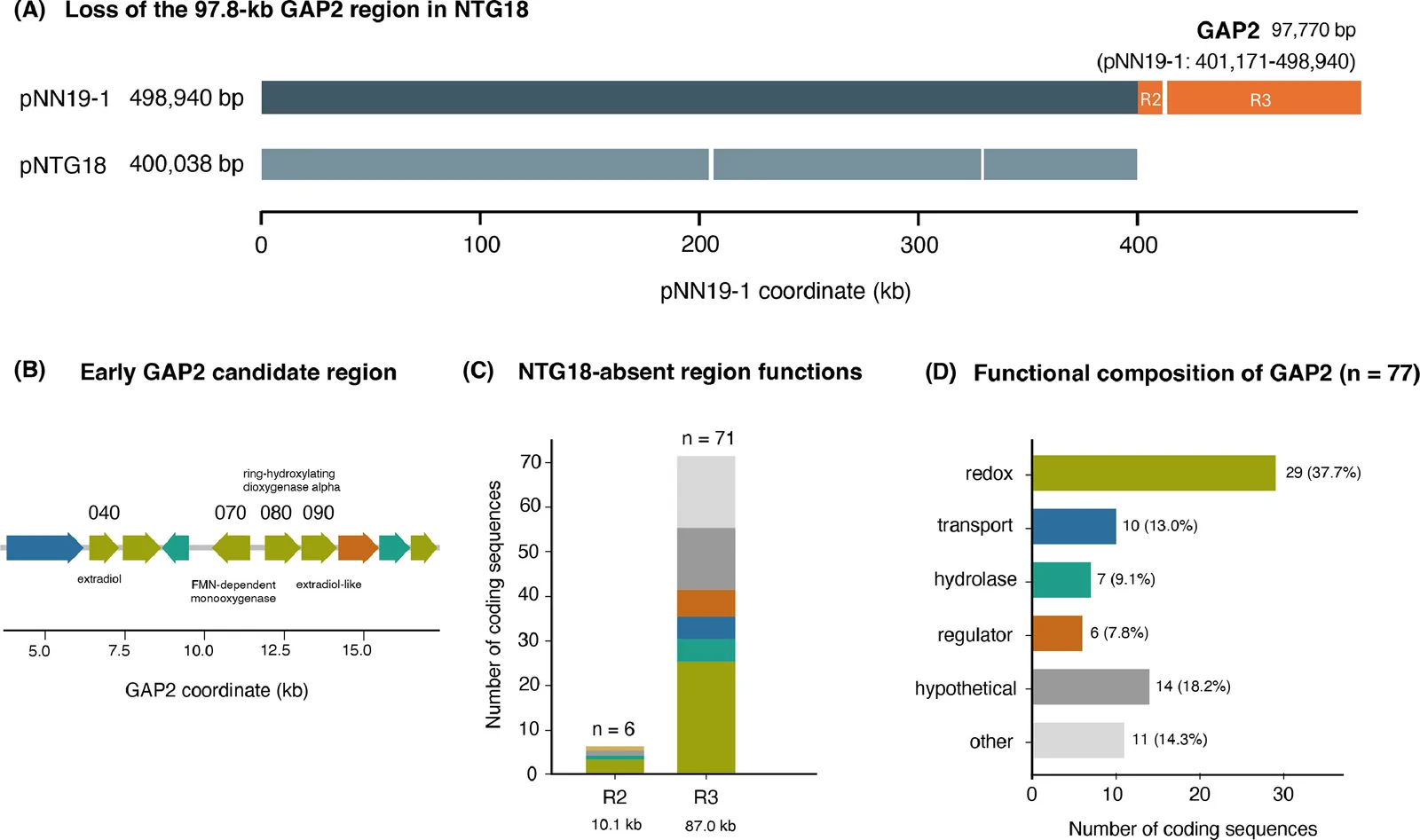
Loss of a plasmid-associated accessory region in the non-transforming NN19-derived mutant NTG18. (A) Linear overview of pNN19–1 showing the GAP2 interval and the R2/R3 regions absent from NTG18. GAP2 corresponds to pNN19–1 positions 401,171 to 498,940 and includes the 10.1-kb R2 region and 87.0-kb R3 region identified through NN19-vs-NTG18 whole-genome alignment. (B) Enlarged map of the early GAP2 candidate region showing LOCUS_040, LOCUS_070, LOCUS_080, and LOCUS_090 together with neighboring accessory genes. Gene arrows indicate coding direction, and colors indicate predicted functional categories. (C) Functional composition of the GAP2-associated pNN19–1 regions R2 and R3 absent from NTG18. (D) Functional composition of the full 97.8-kb GAP2 interval. The co-loss of CPT-transforming activity and a broader plasmid-associated accessory region enriched in redox, hydrolase, transport, and regulatory functions identified GAP2 as the main candidate genomic region associated with CPT transformation. Colors indicate the same functional categories across panels B–D.

Because NTG18 was derived from NN19 and had lost CPT-transforming activity, the co-loss of this activity and the pNN19-1 regions provided a genetic association between GAP2 and CPT transformation. The broader pNN19-1 interval encompassing the deleted R2/R3 regions was therefore designated GAP2. GAP2 corresponded to pNN19-1 positions 401,171 to 498,940 and included the main plasmid segment absent from NTG18. Thus, GAP2 was not defined by annotation alone but primarily by the comparison between the CPT-transforming strain NN19 and its non-transforming derivative NTG18.

Raw HiFi-read validation confirmed the absence of R2 and R3, whereas the retention of chromosomal and pNN19-1 control regions showed robust read support (Supplementary Table S4B). Variant validation identified 23 high-confidence single-nucleotide variants (SNVs) in NTG18 relative to NN19, including 12 missense changes; of these, 19 were specific to NTG18 among the four same-lineage genomes (Supplementary Tables S4C and S4D). None of the affected proteins was annotated as an obvious candidate mediating CPT oxygenation or ring cleavage. However, the functional consequences of the missense changes are unknown, and their direct or indirect contributions to the non-transforming phenotype cannot be excluded.

### 2.6 GAP2 genes encode oxygenation and ring-cleavage candidates

After defining GAP2 via the NN19-NTG18 deletion comparison, we examined DFAST annotations within this region to identify genes consistent with the OP1 metabolite data (Tanizawa et al., 2018). GAP2 included multiple genes that play predicted roles in aromatic oxidation, ring cleavage, hydrolysis, redox metabolism, and transport (Fig. 4, Fig. 5). Four genes (LOCUS_040 [NN19_28260; extradiol dioxygenase], LOCUS_070 [NN19_28290; NtaA/DmoA-family FMN-dependent monooxygenase], LOCUS_080 [NN19_28300; aromatic ring-hydroxylating dioxygenase alpha subunit], and LOCUS_090 [NN19_28310; extradiol-type hypothetical protein]) were designated the core candidate module. Other GAP2 genes, including LOCUS_050 and LOCUS_060 and additional hydrolases, oxidoreductases, transporters, and transcriptional regulators, were retained to provide broader accessory-region context without assigning them to a ranked candidate class.

**Fig. 5:**
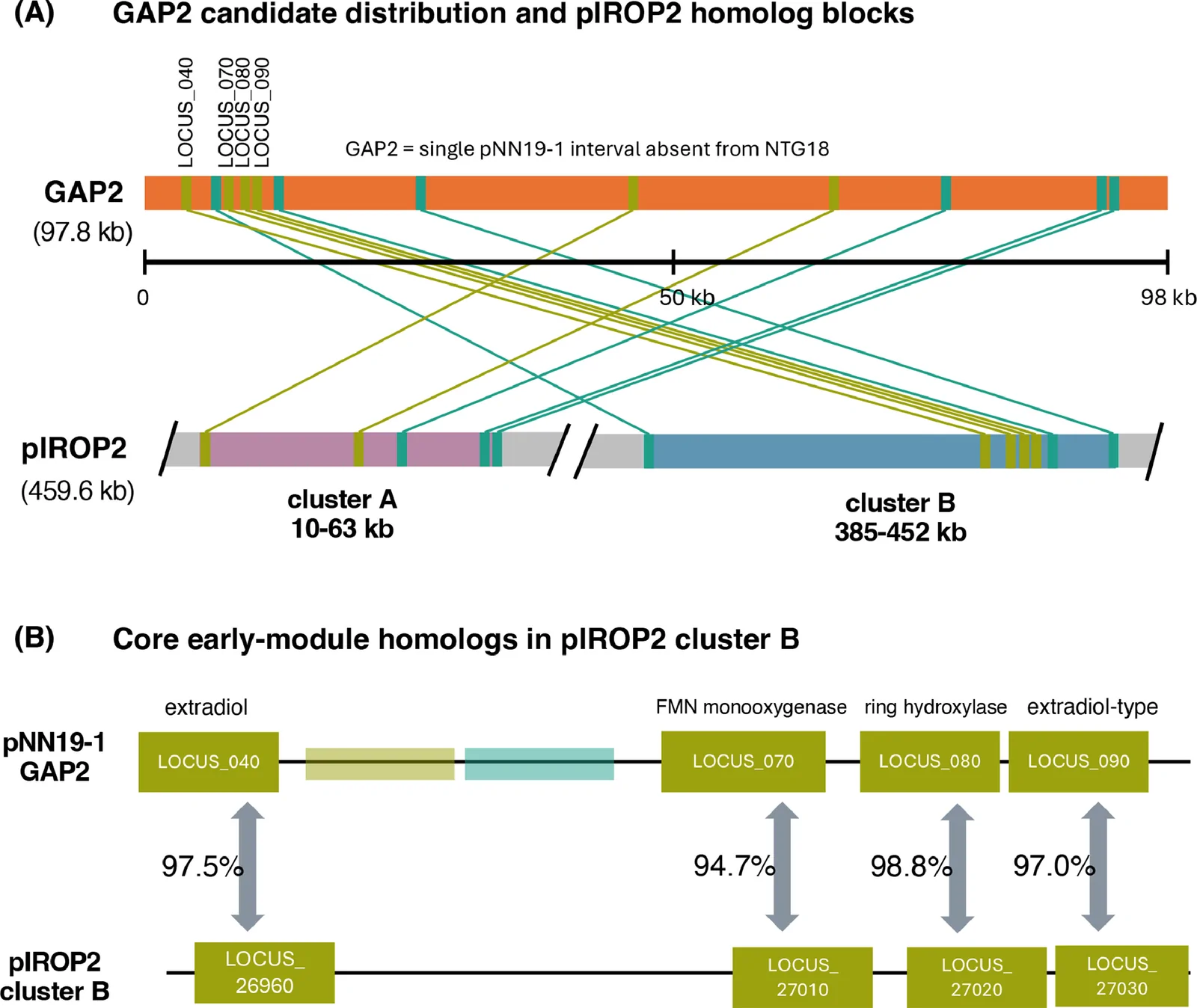
Conservation and rearrangement of GAP2 oxygenation/ring-cleavage candidates in the distinct IROP2 lineage. (A) Comparative map of the NN19 GAP2 candidate region and homologous regions on pIROP2. Colored line segments indicate candidate genes grouped by predicted function; gene direction is omitted in this panel to emphasize regional correspondence. Connecting lines indicate best BLASTp homologs between pNN19–1 and pIROP2, showing that IROP2 carries related homologous blocks in a rearranged plasmid context. (B) Enlarged view of the core early oxidation/ring-cleavage module in pIROP2 cluster B. Homologs include LOCUS_26960 corresponding to NN19 LOCUS_040, LOCUS_27010 corresponding to LOCUS_070, LOCUS_27020 corresponding to LOCUS_080, and LOCUS_27030 corresponding to LOCUS_090. Amino acid identity values are shown for each best hit. Conservation of this compact LOCUS_040/070/080/090 module in a distinct CPT-transforming lineage supports it as a prioritized candidate gene set within the broader GAP2 region. Segments shown in olive, light-olive, and light-teal colors indicate predicted oxidation/ring-cleavage, redox-support, and hydrolase functions, respectively. Gray double-headed arrows connect best BLASTp homologs, and percentages indicate amino acid identity.

Several genes within the early portion of GAP2 were particularly notable, including LOCUS_040, which encodes an extradiol dioxygenase; LOCUS_070, which encodes an NtaA/DmoA-family FMN-dependent monooxygenase; LOCUS_080, which encodes the alpha subunit of an aromatic ring-hydroxylating dioxygenase; and LOCUS_090, which encodes a hypothetical protein annotated as an extradiol dioxygenase candidate. This combination of predicted enzymatic functions matches the chemistry inferred from the OP1 metabolite data: initial oxygenation of the CPT aromatic/polycyclic scaffold, followed by ring-cleavage–type processing.

The NTG18 deletion and GAP2 annotations narrowed the candidate region to a plasmid segment enriched in oxygenation and ring-cleavage functions. However, as GAP2 still included multiple plausible genes, additional evidence will be needed in order to prioritize the most likely responsible gene set.

### 2.7 Accessory-genome analysis placed GAP2 in a broader plasmid-associated accessory-region context

To determine whether GAP2 represents an isolated candidate gene set or is part of a broader accessory region, we re-examined the pNN19-1 regions absent from the non-transforming derivative NTG18 and classified their annotated coding sequences based on predicted function (Figure 4C; Supplementary Tables S5A and S5B). The primary GAP2-associated losses corresponded to pNN19-1 regions R2 and R3. R2 encompassed six coding sequences, including three predicted oxidation-reduction genes and one hydrolase-related gene. R3 encompassed 71 coding sequences and was enriched in predicted oxidation-reduction genes (25 genes), hydrolase-related genes (5 genes), transport or receptor genes (5 genes), and transcriptional regulators (6 genes), in addition to hypothetical and other accessory genes. Thus, the NTG18 deletion did not remove a single isolated candidate gene but rather removed a plasmid-associated region with multiple functions commonly associated with aromatic-compound uptake, oxidation, ring transformation, regulation, and downstream processing.

The 97.8-kb GAP2 interval contained 77 coding sequences, whose functional classifications are summarized in Fig. 4D and Supplementary Table S6. Functional classification assigned 29 genes (37.7%) to oxidation-reduction functions, 7 genes (9.1%) to hydrolase-related functions, 10 genes (13.0%) to transport or receptor functions, and 6 genes (7.8%) to transcriptional regulation. This composition supports the interpretation of GAP2 as a catabolic-island–like accessory region rather than a narrow single-enzyme locus.

The above results indicate that the GAP2 candidate module is embedded within a larger pNN19-1 accessory region that combines oxygenation, redox, hydrolase, transport, and regulatory functions. Because the current data do not demonstrate genetic sufficiency of GAP2, this architecture is best interpreted as a genomic feature associated with CPT-transforming *Sphingobium* isolates and as a prioritized target for expression-based and functional validation.

### 2.8 IROP2 carries a related GAP2-like candidate module

To determine whether the selected deletion-defined GAP2 candidates were conserved beyond the OP1/NN19/KJ09 species-level cluster, we analyzed the distinct CPT-transforming lineage represented by IROP2. BLASTp analysis against the IROP2 DFAST protein set identified homologs of 11 of 12 functionally selected GAP2 candidate proteins (Camacho et al., 2009). These homologs included LOCUS_26960, corresponding to core candidate LOCUS_040 with 97.5% identity and 100% query coverage; LOCUS_27010, corresponding to LOCUS_070 with 94.7% identity; LOCUS_27020, corresponding to LOCUS_080 with 98.8% identity; and LOCUS_27030, corresponding to LOCUS_090 with 97.0% identity. These homologs were located on pIROP2 in two clusters, indicating that IROP2 carries a related but rearranged plasmid-associated module (Figure 5; Supplementary Tables S7A and S7B).

The IROP2 comparison therefore provides evidence of conservation for the core GAP2 candidates across independent CPT-transforming *Sphingobium* lineages. In particular, conservation of LOCUS_080, LOCUS_070, LOCUS_040, and LOCUS_090 supports prioritization of the early oxygenation/ring-cleavage module over genes present only in the broader GAP2 interval.

Consistent with the abovementioned genomic conservation, IROP2 generated oxygenated products from CPT analogs. Time-course HPLC chromatograms are shown in Supplementary Figure S7, and LC-MS analysis detected oxygenated products from both HCPT and EHCPT. One HCPT-derived product gave an ion at *m*/*z* 397.10159, consistent with C_20_H_17_N_2_O_7_ [M+H]^+^ (calculated *m*/*z* 397.10358; mass difference, −1.98 mmu), corresponding to the addition of two oxygen atoms relative to HCPT. Two EHCPT-derived products were also detected: one consistent with C_22_H_21_N_2_O_6_ [M+H]^+^ (observed *m*/*z* 409.14160; calculated *m*/*z* 409.13996; mass difference, 1.63 mmu), corresponding to monooxygenation, and another consistent with C_22_H_21_N_2_O_7_ [M+H]^+^ (observed *m*/*z* 425.13548; calculated *m*/*z* 425.13488; mass difference, 0.60 mmu), corresponding to dioxygenation. These metabolite data provide phenotypic support that the distinct IROP2 lineage carries an active CPT-analog oxygenation capacity consistent with the conserved pIROP2 candidate module.

### 2.9 Prioritization of expressed GAP2 candidates during CPT growth using RNA-seq data

To determine whether the GAP2 candidate genes are expressed during growth with CPT, RNA-seq data obtained from NN19 grown with CPT as the sole added carbon source were analyzed. Because matched CPT-free or alternative-carbon-source libraries were not included, the analysis was used to prioritize genes transcribed under the tested condition rather than to assess CPT-specific induction. Paired-end reads were mapped to the NN19 genome using HISAT2 (Kim et al., 2019) and counted at DFAST-predicted CDS features using featureCounts.

Several GAP2 genes were highly expressed during growth on CPT (Supplementary Figure S8). LOCUS_080 (NN19_28300), which encodes the alpha subunit of an aromatic ring–hydroxylating dioxygenase, showed the highest expression among GAP2 candidates, with a mean HISAT2-featureCounts (Liao et al., 2014) transcripts per million (TPM) of 9316.9 and genome-wide expression rank of 13 among all predicted NN19 CDSs (Table 2). LOCUS_070 (NN19_28290), which encodes an NtaA/DmoA-family FMN-dependent monooxygenase, was also expressed at a high level, with a mean TPM of 1428.5 and rank of 113. LOCUS_040 (NN19_28260), which encodes an extradiol dioxygenase, showed a mean TPM of 1394.8 and rank of 116, and LOCUS_090 (NN19_28310), which encodes an extradiol-type hypothetical protein, showed a mean TPM of 982.6 and rank of 166. Replicate-level TPM values, means, standard deviations, and genome-wide ranks for the selected genes are summarized in Table 2, and the complete GAP2 expression dataset is provided in Supplementary Table S8.

**Table 2:** Expression of selected GAP2 genes during growth of NN19 with camptothecin.

**GAP2 locus**	**NN19 locus**	**Predicted product**	**Candidate class**	**Rep3 TPM**	**Rep4 TPM**	**Rep5 TPM**	**Mean TPM**	**SD TPM**	**Genome-wide expression rank**
LOCUS_040	NN19_28,260	extradiol dioxygenase	core candidate module	1403.3	1431.6	1349.5	1394.8	41.7	116
LOCUS_050	NN19_28,270	NAD(P)/FAD-dependent oxidoreductase	other GAP2 gene	290.5	317.2	305.8	304.5	13.4	583
LOCUS_060	NN19_28,280	amidohydrolase family protein	other GAP2 gene	51.5	27.3	31	36.6	13.1	2501
LOCUS_070	NN19_28,290	NtaA/DmoA family FMN-dependent monooxygenase	core candidate module	1471.8	1423.7	1389.9	1428.5	41.2	113
LOCUS_080	NN19_28,300	aromatic ring-hydroxylating dioxygenase subunit alpha	core candidate module	9691.9	9223.4	9035.3	9316.9	338.2	13
LOCUS_090	NN19_28,310	hypothetical protein (extradiol dioxygenase)	core candidate module	966.2	1029.5	952.2	982.6	41.1	166
LOCUS_100	NN19_28,320	MmgE/PrpD family protein	other GAP2 gene	287.5	292.6	291.9	290.7	2.7	611

*Abbreviations:* FAD, flavin adenine dinucleotide; FMN, flavin mononucleotide; GAP2, genomic accessory region 2; NAD(P), nicotinamide adenine dinucleotide (phosphate); SD, standard deviation; TPM, transcripts per million.

The expression pattern highlighted a compact early oxidation/ring-cleavage module within GAP2. The most highly expressed candidate, LOCUS_080, is predicted to catalyze aromatic ring hydroxylation or dioxygenation, whereas LOCUS_070 may encode the enzyme for a complementary FMN-dependent monooxygenation step. LOCUS_040 and LOCUS_090 are predicted to encode extradiol-type enzymes that may function after initial oxygenation to cleave or rearrange oxygenated CPT intermediates. By contrast, LOCUS_060, which encodes an amidohydrolase-family protein, showed much lower transcript abundance. These results prioritize GAP2 candidates transcribed during growth with CPT.

### 2.10 A working hypothesis for bacterial CPT transformation

Integrating the metabolite, NTG18 deletion, IROP2 conservation, annotation, and RNA-seq data led to a working hypothesis in which entry into CPT metabolism begins with oxygenation of the CPT scaffold followed by ring cleavage. In this hypothesis, LOCUS_080 is a primary candidate for mediating an early oxygenation step because it encodes the alpha subunit of an aromatic ring-hydroxylating dioxygenase, is conserved in the pIROP2 homologous module, and is the most highly expressed GAP2 candidate during growth on CPT. LOCUS_070, which encodes an NtaA/DmoA-family FMN-dependent monooxygenase, may act in parallel or sequentially to introduce oxygen into CPT or an early CPT-derived intermediate.

After initial oxygenation, the products of LOCUS_040 and LOCUS_090 are plausible candidates for mediating ring-cleavage reactions. LOCUS_040 is annotated as an extradiol dioxygenase, whereas LOCUS_090 is annotated as a hypothetical protein with extradiol-dioxygenase features. These enzymes are therefore more likely to act on hydroxylated or catechol-like CPT-derived intermediates than directly on unmodified CPT.

Additional GAP2 genes may support this early pathway. LOCUS_050, which encodes an NAD(P)/FAD-dependent oxidoreductase, may provide redox support for oxygenase activity or downstream intermediate processing. LOCUS_060 and other genes within the broader GAP2 region that encode hydrolase-like enzymes may participate in downstream processing of CPT-derived products. This organization is consistent with the transient formation and subsequent turnover of M1 and with the substrate-selective oxygenative transformation observed in the HCPT and EHCPT assays.

Although direct biochemical validation of individual enzymes is needed, the convergence of four independent lines of evidence supports the GAP2 module as the leading candidate genomic region associated with bacterial CPT transformation, as follows: (i) the non-transforming NN19-derived mutant NTG18 lacks the primary GAP2 region; (ii) the distinct CPT-transforming IROP2 lineage carries a related pIROP2 module with homologs of the core early oxygenation/ring-cleavage candidates; (iii) the core oxygenase/ring-cleavage genes are highly expressed during CPT growth; and (iv) the predicted enzyme functions are consistent with the observed formation and turnover of CPT-derived products.

## 3 Discussion

The results of this study provide a combined phenotypic, chemical, genomic, and transcriptomic framework to explain the bacterial metabolism of the medically important plant alkaloid CPT (Hartmann and Lipp, 2006; Hsiang et al., 1985; Mathijssen et al., 2002; Wall et al., 1966). Although CPT has been extensively studied as a natural product scaffold for anticancer drugs and as a plant secondary metabolite, microbiological studies have primarily focused on CPT production by endophytes or on biotechnological supply rather than microbial transformation or turnover (El-Hady et al., 2024; Soujanya et al., 2017). Environmental studies have examined abiotic degradation and risk, but such studies have reported negligible biodegradation in seawater (Hao et al., 2022). Thus, bacterial transformation and metabolic entry of CPT in plant-associated environments have remained largely unexplored. By isolating CPT-transforming *Sphingobium* strains, characterizing transient CPT-derived metabolites, and using a non-transforming NN19-derived mutant to define a plasmid-associated candidate region, this study connects the environmental fate of CPT with specific bacterial lineages and candidate genetic determinants.

### 3.1 CPT metabolism as a plant-rhizosphere microbial function

The isolation of OP1 from the rhizosphere of the CPT-producing plant *Ophiorrhiza pumila* is consistent with the capacity of plant-associated microorganisms to utilize or transform specialized plant metabolites (Jilani et al., 2008; Lee et al., 2020; Nakayasu et al., 2023; Pang et al., 2024; Rathbone and Bruce, 2002; Sudo et al., 2002; Sugiyama, 2023; Yamazaki et al., 2007). OP1 transformed CPT in resting-cell assays and produced a transient product M1. M1 retained the E-ring lactone, and neither marked CPT depletion nor M1 formation occurred in the cell-free control. CPT also remained near its initial abundance during incubation with three non-transforming reference *Sphingobium* strains, while the bulk culture pH in the growth experiment remained essentially unchanged at 7.31-7.33. These observations indicate that the results cannot be explained solely by pH-dependent hydrolysis of the CPT E-ring lactone. The ability of OP1 to transform HCPT, together with its delayed or limited transformation of EHCPT, further suggests that CPT metabolism is substrate selective. This selectivity is consistent with enzyme-mediated recognition of the CPT scaffold and suggests that either the position or the steric environment of substituents on CPT analogs can affect bacterial transformation.

These findings are relevant in the contexts of both chemical ecology and biocatalysis. In the rhizosphere, bacterial transformation can affect the persistence, toxicity, and biological activity of CPT released by plants (Jilani et al., 2008; Lee et al., 2020; Nakayasu et al., 2023; Pang et al., 2024; Sudo et al., 2002; Sugiyama, 2023; Yamazaki et al., 2007). The transient accumulation and subsequent disappearance of M1 indicate that OP1 further transforms M1 rather than accumulating it as a stable end product. Therefore, plant-associated CPT-transforming *Sphingobium* strains may reduce the persistence of bioactive CPT-derived structures in root-associated environments, although direct comparisons of toxicity between CPT and downstream metabolites remain to be performed. This interpretation places CPT metabolism in the broader context of microbial adaptation to plant-derived bioactive nitrogenous compounds, as has been described for microbial degradation of caffeine and nicotine (Lin et al., 2023; Zhang et al., 2022). From a biocatalytic perspective, the formation of M1 and oxygenated CPT-analog products suggests that CPT-transforming bacteria express enzymes capable of selectively transforming complex alkaloid scaffolds (Barry and Challis, 2013; Fetzner, 2012; Kweon et al., 2008; Lipscomb, 2008; Pati et al., 2022; Rathbone and Bruce, 2002; Xu et al., 1997). The identification of candidate genes therefore provides a starting point for future enzymatic and synthetic biology–related studies.

### 3.2 CPT-transforming isolates represent previously undescribed *Sphingobium* lineages

TYGS analyses placed OP1, NN19, and KJ09 in a single genome species-level cluster and IROP2 in a closely related but distinct species-level lineage. Thus, the strain set represents two related CPT-transforming *Sphingobium* lineages. OP1 provides the primary metabolite evidence, NN19 provides the primary genome and RNA-seq evidence, KJ09 provides additional support within the same lineage, and IROP2 tests whether the candidate module is conserved beyond the OP1/NN19/KJ09 group.

The previously undescribed status of these isolates is important because CPT transformation is not being assigned to a previously described model degrader or a known species with an established CPT-transformation phenotype. To the best of our knowledge, this study reports the first isolation and genomic characterization of bacterial lineages that metabolize CPT. Our results therefore expand CPT microbiology beyond the familiar theme of endophytic CPT production and place bacterial CPT transformation in a plant-associated ecological context.

Relative to available type-strain genomes, TYGS identified the OP1/NN19/KJ09 cluster and IROP2 lineage as potentially novel species-level lineages (Meier-Kolthoff and Göker, 2019). A formal description of new bacterial species would require additional taxonomic work, including valid publication, deposition of the type strain in public culture collections, and phenotypic characterization (de Lajudie et al., 2019; Parker et al., 2019). Therefore, the present study conservatively treats these lineages as putative, novel, species-level lineages rather than formally described new species. Even without formal species descriptions, their placement is biologically important because it shows that CPT transformation is associated with previously uncharacterized *Sphingobium* diversity in plant-associated environments.

This placement is also consistent with a broader feature of *Sphingobium* biology. Characterized members of the genus and related sphingomonads often harbor accessory replicons that encode catabolic functions for chemically specialized substrates, including aromatic and xenobiotic compounds, with UT26 providing a classic example of the distribution of catabolic genes on multiple replicons (Nagata et al., 2006, 2007, 2010; Romine et al., 1999; Stolz, 2014). SYK-6 provides a complementary example in which a *Sphingobium* strain is highly specialized for the metabolism of lignin-derived aromatic compounds (Masai et al., 2012), whereas BD3100 shows that *Sphingobium* can also transform and utilize plant-derived protoberberine alkaloids (Takeda et al., 2015). Plant-associated *Sphingobium* strains have also been linked to the degradation of plant allelochemicals, including pterostilbene and phenolic acids (Yu et al., 2019; Zhang et al., 2024). The present isolates extend this genomic and ecological theme to the scaffold of CPT, a structurally distinct plant alkaloid. Thus, the taxonomic result represents more than a classification step; it suggests that plant-associated *Sphingobium* lineages function according to the same accessory-genome logic that underlies other sphingomonad catabolic phenotypes in order to utilize specialized plant metabolites.

### 3.3 A plasmid-associated accessory region links genome architecture to CPT transformation

The NN19-NTG18 comparison defined GAP2, a plasmid-associated region on pNN19-1 that is absent from the non-transforming mutant, as the primary candidate region associated with CPT transformation. DFAST-based annotation of this deletion-defined region showed that it contains genes encoding enzyme families that match the chemistry inferred from metabolite analyses, including aromatic ring-hydroxylating dioxygenases, FMN-dependent monooxygenases, extradiol-type dioxygenases, oxidoreductases, hydrolases, transporters, and transcriptional regulators. Such an organization is consistent with pathways for aromatic-compound degradation, in which initial oxygenation activates an aromatic substrate for subsequent ring cleavage and downstream processing (Barry and Challis, 2013; Fetzner, 2012; Kweon et al., 2008; Lipscomb, 2008; Pati et al., 2022; Xu et al., 1997). It also places the core oxygenation/ring-cleavage candidates within a broader accessory-region architecture rather than within a single-gene model.

The NTG18 comparison defines GAP2 as the primary candidate region associated with CPT transformation. NTG18 is a non-transforming mutant derived from NN19 and lacks the major GAP2-containing region of pNN19-1, including LOCUS_080, LOCUS_070, LOCUS_040, LOCUS_090, and neighboring redox, hydrolase, transport, and regulatory candidates. The comparable growth of NTG18 under nonselective conditions and the absence of an obvious core-genome loss argue against a broad fitness defect as the primary cause of the phenotype. HiFi-read analysis identified additional high-confidence SNVs in NTG18, including missense changes in regulatory, stress-response, and metabolic proteins (Supplementary Tables S4C and S4D). None were annotated as obvious CPT oxygenation or ring-cleavage candidates, although indirect contributions cannot be excluded. Thus, the co-loss of CPT-transforming activity and GAP2 provides a loss-of-function association that is strengthened by functional annotation, conservation in IROP2, and expression during growth with CPT.

GAP2 comprises 77 coding sequences and is enriched in predicted oxidation-reduction functions, together with hydrolase-related, transport or receptor, and transcriptional-regulatory genes. This architecture identifies GAP2 as a plasmid-associated accessory module with the biochemical breadth expected for substrate uptake, oxidative activation, ring transformation, regulation, and downstream processing.

The detection of homologous GAP2 candidate genes on pIROP2 further suggests that the module is not limited to one strain or one species-level lineage. IROP2 is genomically distinct from OP1/NN19/KJ09 but carries high-identity homologs of the four core candidates (LOCUS_080, LOCUS_070, LOCUS_040, and LOCUS_090). This conservation strengthens prioritization of the core candidate module relative to other genes found only in a single plasmid context. The pIROP2 arrangement appears related but not identical to GAP2, suggesting conservation of a candidate functional signature with rearrangement or divergence. The detection of oxygenated HCPT and EHCPT metabolites in IROP2 further supports an association between this homologous module and CPT-analog oxygenation activity in a distinct species-level lineage.

### 3.4 Oxygenation followed by ring cleavage is the most plausible current pathway model

The combined evidence supports an oxygenation-first model for entry into CPT metabolism. LOCUS_080, which encodes the alpha subunit of an aromatic ring–hydroxylating dioxygenase, is currently a primary candidate for an early oxygenation step. This assignment is supported by enzyme family, predicted chemistry, conservation in the distinct pIROP2 module, absence from NTG18, and especially by high expression during growth on medium containing CPT. LOCUS_070, which encodes an NtaA/DmoA-family FMN-dependent monooxygenase, is also a plausible candidate and may act either in parallel with LOCUS_080 or on an early oxygenated CPT intermediate (Barry and Challis, 2013; Kweon et al., 2008; Pati et al., 2022; Xu et al., 1997).

LOCUS_040 and LOCUS_090 are better interpreted as ring-cleavage or post-oxygenation enzymes than as primary first-attack enzymes. The extradiol-type annotations of these genes are consistent with possible cleavage of hydroxylated or catechol-like aromatic intermediates (Fetzner, 2012; Lipscomb, 2008). This interpretation is consistent with the transient behavior of M1 in the HPLC time course: M1 accumulated during the early phase and subsequently decreased, suggesting further downstream transformation rather than accumulation as a terminal product. The products of LOCUS_050 and LOCUS_060 may support the pathway through redox and hydrolytic reactions, respectively, although the exact roles of these genes remain to be determined.

The RNA-seq results are consistent with this model. The prioritized oxygenation and ring-cleavage candidates were transcribed during growth with CPT, while the resting-cell assays showed comparable CPT-transforming activity in cells precultured with and without CPT, indicating that detectable activity does not require strong prior substrate induction under the tested conditions. Because matched CPT-free or alternative-carbon-source libraries were not included, the RNA-seq data are interpreted as transcriptional support for candidate prioritization rather than evidence of CPT-specific regulation.

### 3.5 Limitations and future directions

The primary limitation of the present study is that the functions of individual candidate enzymes have not yet been genetically or biochemically demonstrated. Targeted genetic manipulation was attempted in these newly isolated *Sphingobium* strains using pK18*mobsacB*-, pAK405-, and pJB861-based approaches, including electroporation and conjugative transfer, but reproducible gene disruption or complementation was not obtained under the tested conditions. Heterologous expression may require codon-optimized synthetic genes and appropriate partner proteins or cofactors. Genetic complementation and biochemical characterization of the prioritized enzymes will therefore be required to assign individual reaction steps within the proposed module.

Several experiments would strengthen the pathway model. More complete metabolite comparisons between IROP2 and OP1, especially for CPT itself, would determine whether the distinct IROP2 lineage produces the same CPT-derived products or a divergent metabolite profile. Recombinant expression of selected candidates, particularly LOCUS_080 and LOCUS_070, could allow testing of whether they transform CPT or CPT-derived intermediates in the presence of appropriate redox partners (Barry and Challis, 2013; Kweon et al., 2008; Pati et al., 2022; Xu et al., 1997). LC-MS–based metabolite profiling of wild-type NN19 and NTG18 could directly connect loss of GAP2 to loss of specific CPT-derived metabolites. Development of genetic tools for these isolates would enable targeted deletion or complementation of the candidate module.

Despite these limitations, the convergence of metabolite chemistry, genome-based taxonomy, loss of GAP2 in the non-transforming mutant NTG18, accessory-genome architecture, conservation of core candidates in IROP2, and transcription during growth with CPT provides a coherent genetic framework for bacterial CPT metabolism. These findings support a testable model in which a plasmid-associated oxygenation/ring-cleavage module enables plant-associated *Sphingobium* lineages to enter the metabolism of a structurally complex plant alkaloid. More broadly, they raise the ecological hypothesis that specialized plant metabolites can act as selective chemical factors favoring bacteria that carry accessory metabolic functions capable of transforming these compounds.

## 4 Conclusion

This study identified plant-associated *Sphingobium* lineages that transform CPT and linked this phenotype to the plasmid-associated GAP2 region. Strain OP1 formed the transient product M1, which retained the E-ring lactone and subsequently decreased during incubation, and its viable counts increased as CPT was depleted when CPT was supplied as the sole added carbon source. Loss of GAP2 in the non-transforming mutant NTG18, conservation of a rearranged homologous module on pIROP2, and transcription of prioritized genes during growth with CPT converge on a compact oxygenation/ring-cleavage candidate module. Together, these findings support a testable model in which plasmid-associated accessory metabolism enables bacterial entry into the metabolism of a structurally complex plant alkaloid and may contribute to adaptation in chemically selective plant-associated habitats. The prioritized genes provide focused targets for genetic and biochemical validation of the individual reaction steps.

## 5 Materials and methods

### 5.1 Bacterial strains, media, and chemicals

The CPT-transforming isolates and NN19-derived mutant used in genome-based analyses are listed in Table 1. The CPT-transforming isolates OP1, NN19, and KJ09 were obtained from plant-associated soils collected at the medicinal plant garden of Hoshi University, Tokyo, Japan. IROP2 was isolated from soil associated with *Ophiorrhiza pumila* Champ. ex Benth. collected near the upper Urauchi River on Iriomote Island, Okinawa, Japan. NTG18 was used as a non–CPT-transforming mutant derived from NN19. Reference *Sphingobium* strains used for comparisons included *S. japonicum* UT26, *S. chlorophenolicum* ATCC 33790, and *S. yanoikuyae* ATCC 51230.

Bacteria were cultivated in LB medium consisting of 10 g of Bacto tryptone, 5 g of Bacto yeast extract, and 5 g of NaCl per liter of distilled water, or in WM inorganic salts medium, which consisted of 1.7 g of KH_2_PO_4_, 12.4 g of Na_2_HPO_4_·12H_2_O, 1.0 g of (NH_4_)_2_SO_4_, 2.0 mg of CaCO_3_, 1.4 mg of ZnSO_4_·7H_2_O, 0.25 mg of CuSO_4_·5H_2_O, 0.28 mg of CoSO_4_·7H_2_O, 0.06 mg of H_3_BO_4_, 100 mg of MgSO_4_·7H_2_O, 0.95 mg of FeSO_4_·7H_2_O, and 10.7 mg of MgO per liter. A 0.1 × LB medium was prepared by reducing the Bacto tryptone and Bacto yeast extract concentrations of LB medium to one-tenth. R2A agar medium was purchased from Becton, Dickinson and Company (Franklin Lakes, NJ, USA). For solid media, agar was added at 1.5% (w/v). Kanamycin was added at a final concentration of 50 µg/mL where required. CPT, HCPT, and EHCPT were dissolved in DMSO at 5 mg/mL and added to media at 1% (v/v) or to assay mixtures as described below. Cultures were incubated at 30 °C, and liquid cultures were shaken at 180 rpm unless otherwise stated.

For routine liquid cultivation, KJ09, NN19, OP1, IROP2, and NTG18 were inoculated into 3 mL of LB medium and precultured at 30 °C for 3 days. The reference strains *S. chlorophenolicum, S. japonicum*, and *S. yanoikuyae* were grown overnight in 3 mL of LB medium at 30 °C. KJ09, NN19, OP1, IROP2, and NTG18 were incubated on LB agar plates at 30 °C for 5–7 days after streaking. These strains were incubated at 30 °C for 7–10 days when cultured on WM agar plates containing CPT, HCPT, or EHCPT.

### 5.2 Enrichment and isolation of CPT-transforming bacteria

Soil samples were collected from plant-associated environments for enrichment of CPT-transforming bacteria. At the medicinal plant garden of Hoshi University, 5 *g* soil samples were collected from near the roots of *Camptotheca acuminata* Decne., *Nothapodytes nimmoniana* (J.Graham) Mabb., and *O. pumila* plants. On Iriomote Island, *g*5-g soil samples were collected from near the roots of wild *O. pumila* plants growing near the upper Urauchi River (under permit no. 2503243, issued by the Kyushu Regional Environment Office, Ministry of the Environment, Government of Japan).

For enrichment, 0.5 g of soil was added to 100 mL of WM medium containing 100 mg of CPT and incubated at 30 °C for 5 days with shaking at 180 rpm. A 1-mL aliquot was then transferred to fresh WM medium containing CPT and incubated again under the same conditions. This transfer was repeated five times. Enriched cultures were streaked onto CPT-containing WM agar or CPT-containing 0.1 × LB agar, and single colonies were isolated after incubation. Single colonies were picked with sterile toothpicks, cultured in 3 mL of LB medium at 30 °C for 3 days, mixed with glycerol, and stored at −80 °C. CPT-derived fluorescence on agar plates was monitored by illumination with a 365-nm UV-LED black light (PW-UV343H-03 L; Contec Co., Ltd., Osaka, Japan) as a qualitative screen for local CPT depletion or transformation.

### 5.3 16S rRNA gene sequencing and initial taxonomic identification

For initial identification, OP1 was grown in LB medium at 30 °C for 4 days with shaking. Total genomic DNA was extracted from bacterial cell pellets using a Genomic DNA Purification kit (Promega K.K., Tokyo, Japan) according to the manufacturer’s protocol. The 16S rRNA gene was amplified using primers 27F (5′-GAG TTT GAT CCT GGC TCA G-3′) and 1525R (5′-GAG GTG ATC CAG CCG CAG G-3′). PCR amplification was performed with KOD One DNA polymerase (Toyobo Co., Ltd., Osaka, Japan) under the following conditions: initial denaturation at 96 °C for 120 s; 32 cycles of 98 °C for 10 s, 55 °C for 5 s, and 68 °C for 10 s.

Amplified products were purified using a FastGene Gel/PCR Extraction kit (Nippon Genetics Co., Ltd., Tokyo, Japan), cloned into the *Sma*I site of pUC19, and sequenced using the BigDye Terminator Cycle Sequencing system (Applied Biosystems, Foster City, CA, USA) on an ABI 3100 automated sequencer. Sequencing primers included M13–20, 520F (5′-CAG CCG CGT AAT AC-3′), 520R (5′-GTA TTA CCG CGG CTG-3′), 920F (5′-AAA CTC AAA TGA ATT GAC GG-3′), 920R (5′-CCG TCA ATT CAT TTG AGT TT-3′), and M13-Rv. The resulting 16S rRNA gene sequences were compared with sequences deposited in NCBI using BLAST+ (Camacho et al., 2009).

### 5.4 Growth and CPT depletion in inorganic salts medium

For CPT depletion assays, 3-mL LB precultures of OP1, *Sphingobium japonicum* UT26, *S. chlorophenolicum* ATCC 33,790, and *S. yanoikuyae* ATCC 51230 were washed twice with WM inorganic salts medium and resuspended in 3 mL of the same medium. Washed cell suspension (0.5 mL) was inoculated into three independent 50-mL WM cultures containing 5 mg of CPT and incubated at 30 °C with shaking at 180 rpm. At 0, 12, 24, 36, 48, 60, and 72 h, 0.5-mL samples were mixed with an equal volume of methanol, centrifuged, and analyzed by HPLC. CPT peak areas were normalized to the corresponding 0-h values, and data are presented as the mean ± standard deviation (SD; n = 3).

Because suspended CPT precluded reliable optical-density measurements, OP1 growth was determined by viable counts. A 4-mL LB preculture was washed twice with WM medium, resuspended in 4 mL, and inoculated (0.5 mL) into three independent 50-mL WM cultures with 5 mg of CPT and three CPT-free cultures. Cultures were incubated as above. At 0, 12, 24, 48, 72, 96, and 120 h, 0.5-mL samples were serially diluted tenfold in WM medium, and 100-µL aliquots were spread onto R2A agar and incubated at 30 °C for 4 days. Counts were expressed as log10 colony-forming units per milliliter (CFU/mL; mean ± SD, n = 3); the detection limit was 100 CFU/mL. For pH measurements, 5-mL samples collected at 0, 48, and 120 h were centrifuged, and the supernatant pH was measured using an MP220 pH meter (Mettler Toledo, Greifensee, Switzerland). Values are presented as the mean ± SD of three independent cultures.

### 5.5 NTG mutagenesis and selection of the non–CPT-transforming mutant NTG18

NN19 was mutagenized with *N*-methyl-N'-nitro-N-nitrosoguanidine (NTG) to isolate mutants defective in CPT-transforming activity. NN19 was precultured in LB medium at 30 °C for 3 days, and 1 mL of the preculture was inoculated into 100 mL of LB medium and incubated for 24 h at 30 °C with shaking. Cells were harvested by centrifugation at 8310 × *g* for 15 min, washed twice with distilled water, and suspended in 1 mL of TM buffer (0.5 mM Tris–HCl [pH 7.5], 10 mM MgSO_4_). NTG solution (5 mg/mL in TM buffer) was added at 0, 100, 200, or 500 µL, and the resulting suspensions were incubated at 30 °C for 30 min. The cells were then collected by centrifugation at 10,000 rpm for 1 min, washed with 1 mL of LB medium, and then 300 µL of the suspension was inoculated into 3 mL of LB medium for overnight recovery at 30 °C. Cultures were diluted 10^3^- to 10^5^-fold, plated on LB agar, and incubated at 30 °C for 7 days. Colonies from NTG treatments exhibiting a > 90% killing rate were replica-streaked onto LB agar and inorganic salts agar containing CPT. Clones that grew on LB agar but failed to grow on CPT-containing inorganic salts agar were selected as candidate non-transforming mutants.

The non–CPT-transforming phenotype of NTG18 was confirmed based on the results of three assays: lack of growth on CPT-containing inorganic salts medium, absence of CPT-derived fluorescence loss on CPT-containing R2A plates, and lack of CPT transformation in HPLC-based resting-cell assays. Growth of NTG18 under nonselective conditions was comparable to that of wild-type NN19.

### 5.6 Resting-cell assays of CPT and CPT analog transformation

Resting-cell assays were performed to evaluate transformation of CPT and related analogs. For comparisons among CPT-transforming isolates, NTG18, and reference strains, all strains were assayed under the same resting-cell conditions, unless otherwise indicated. Bacteria were precultured in 3 mL of LB medium for 2 days at 30 °C. For the comparison with prior CPT exposure, 1 mL of the preculture was inoculated into 100 mL of LB medium or 100 mL of LB medium containing 10 mg of CPT in a 500-mL Erlenmeyer flask and incubated for 3 days at 30 °C with shaking at 180 rpm. After incubation, the cells were harvested, washed twice with phosphate buffer, and suspended in 10 mL of phosphate buffer to an optical density at 600 nm of 2.0. CPT stock solution (100 µL of 5 mg/mL CPT in DMSO) was added to the cell suspension, and the mixture was incubated at 30 °C. At each time point, 0.5-mL aliquots were removed, and the reactions were stopped by adding 0.5 mL of methanol. After centrifugation, the supernatant was analyzed using HPLC. Cell-free reaction mixtures containing CPT but no bacterial cells and reaction mixtures containing the non-transforming reference strains *S. japonicum, S. chlorophenolicum*, or *S. yanoikuyae* served as controls. No M1 peak was detected in the cell-free control by photodiode-array HPLC.

For CPT analog assays, HCPT and EHCPT were tested under the same or analogous resting-cell conditions. In agar-based transformation screening assays, WM agar plates containing HCPT or EHCPT were streaked with OP1 and incubated at 30 °C, and changes in fluorescence were monitored under 365-nm ultraviolet illumination. OP1 and IROP2 resting-cell reaction supernatants from HCPT and EHCPT assays were analyzed using LC-ESI-TOF-MS to determine accurate masses of CPT analog–derived products.

### 5.7 HPLC analysis of CPT, HCPT, and EHCPT transformation

CPT, HCPT, and EHCPT in reaction supernatants were semi-quantitatively analyzed using photodiode array HPLC with a PU-2080 pump (JASCO Co., Ltd., Tokyo, Japan) equipped with an MD-4017 photodiode array detector (JASCO) and an Inertsil ODS-3 column (5 µm, 4.6 × 250 mm; GL Sciences Inc., Tokyo, Japan). The column temperature was maintained at 40 °C using a CO-2065 Plus column oven (JASCO). The flow rate was 1.0 mL/min. Solvent A was 0.1% trifluoroacetic acid in water, and solvent B was acetonitrile. Components were eluted using a gradient consisting of 15% B initially, followed by a linear increase to 85% B from 0 to 15 min, holding at 85% B until 17 min, then returning to the initial condition at 17.1 min and maintenance of the initial condition until 20 min. The eluate was monitored at 254 nm unless otherwise indicated.

### 5.8 Isolation and structural analysis of CPT metabolite M1

For isolation of the CPT metabolite M1, OP1 was grown at 30 °C with shaking for 3 days in 100 mL of LB medium. The cells were washed twice with distilled water and then suspended in 10 mL of distilled water. CPT (5 mg) was added to 200 mL of distilled water and sonicated briefly to form a suspension, after which 10 mL of cell suspension was added. This mixture was incubated at 30 °C with shaking at 180 rpm for 5 h. The cells were then removed by centrifugation and filtration, and the supernatant was concentrated by evaporation to obtain a crude extract. The dried sample was resuspended in 40% methanol and analyzed using HPLC.

Preparative purification was performed on an HPLC system consisting of an InertSustain C18 column (5 µm, 10 × 250 mm; GL Sciences), LC-20AT Prominence pump (Shimadzu Corp., Kyoto, Japan), SPD-20A UV detector (Shimadzu), and CO-965 column oven (JASCO). The column temperature was maintained at 40 °C. The flow rate was 1.0 mL/min, and the eluate was monitored at 254 nm for detection. Initial fractionation was performed using 40% methanol as the mobile phase. Three fractions were collected, and the second fraction was further purified using 35% methanol as the mobile phase. This procedure yielded 3.4 mg of purified M1.

All molecular weight and molecular formula determinations, including analyses of OP1 and IROP2 reaction supernatants, were performed using LC-ESI-TOF-MS with a 1200 series LC system (Agilent Technologies, Santa Clara, CA, USA) equipped with an InertSustain C18 column (3 µm, 2.1 × 250 mm; GL Sciences) and operated at a flow rate of 0.2 mL/min and column temperature of 30 °C. The mobile phase comprised 0.1% formic acid in water (solvent A) and 0.1% formic acid in methanol (solvent B) and was delivered isocratically at 60:40 (A:B [v/v]). The purified compound was further analyzed using nuclear magnetic resonance (NMR) spectroscopy and circular dichroism (CD) spectroscopy for structural characterization. ^1^H- and ^13^C-NMR spectra were recorded on a Bruker AVANCE-400 spectrometer (400.13 MHz for ^1^H- and 100.61 MHz for ^13^C-NMR; Bruker BioSpin K.K., Kanagawa, Japan), and chemical shifts are reported in ppm using tetramethylsilane as an internal standard. Two-dimensional NMR spectra, including HMQC, ^1^H–^1^H COSY, and HMBC, were used for signal assignment and connectivity analysis; NMR assignments are provided in Supplementary Table S3. CD spectra were recorded using a JASCO spectropolarimeter (JASCO).

### 5.9 Genome sequencing, assembly, and annotation

Genomic DNA was extracted from 1 mL of an LB culture using a Blood & Cell Culture DNA Mini kit and Genomic-tip 20/G (Qiagen K.K., Tokyo, Japan) according to the bacterial DNA extraction protocol in the QIAGEN Genomic DNA Handbook. DNA concentrations were determined using a Qubit dsDNA BR assay kit and Qubit 2.0 fluorometer (Thermo Fisher Scientific, Waltham, MA, USA).

For NN19 and KJ09 short-read sequencing, DNA was quantified using a Synergy LX plate reader (BioTek Instruments, Winooski, VT, USA) and QuantiFluor dsDNA System (Promega K.K., Tokyo, Japan). DNA quality was assessed using a 5200 Fragment Analyzer System with an Agilent HS Genomic DNA 50-kb kit (Agilent Technologies). Libraries were prepared using a MGIEasy FS DNA Library Prep Set according to the manufacturer’s protocol, with a 4-min enzymatic fragmentation step and MGIEasy DNA Adapters-96 (Plate) kit adapters. Library concentrations were determined using a Qubit 3.0 fluorometer and Qubit dsDNA HS assay kit (Thermo Fisher Scientific), and library quality was assessed using a Fragment Analyzer with a dsDNA 915 reagent kit (Advanced Analytical Technologies, Inc., Ankeny, IA, USA). Circularized DNA was prepared using a MGIEasy circularization kit, whereas DNBs were prepared using a DNBSEQ-G400RS high-throughput sequencing kit, and sequencing was performed on a DNBSEQ-G400 instrument (MGI Tech Co., Ltd., Tokyo, Japan) in 2 × 200 bp mode.

For NN19 and KJ09 long-read sequencing, libraries were prepared using a Ligation Sequencing kit (Oxford Nanopore Technologies, Oxford, UK), quantified using a Qubit 3.0 fluorometer and dsDNA HS assay kit (Thermo Fisher Scientific), and sequenced on a GridION instrument with an R9.4.1 flow cell. Base calling and barcode demultiplexing were performed in real time using Guppy v4.0.11. For NN19, short-read and long-read sequencing data were assembled using a hybrid assembly workflow with Unicycler v0.5.0.

For OP1, IROP2, and NTG18, DNA quality was assessed using a 5200 Fragment Analyzer System with an Agilent HS Genomic DNA 50-kb kit (Agilent Technologies). DNA was purified using 1.8 vol of DNA Clean Beads (MGI Tech Co., Ltd., Tokyo, Japan) and sheared to approximately 10–20 kbp using a g-TUBE device (Covaris, Woburn, MA, USA). HiFi libraries were prepared using a SMRTbell Express Template Prep kit 2.0 (Pacific Biosciences, Menlo Park, CA, USA) according to the manufacturer’s low-input HiFi library preparation protocol. Polymerase complexes were formed using Binding Kit 2.2 (Pacific Biosciences), and sequencing was performed using a Sequel IIe instrument (Pacific Biosciences). SMRT Link v11.0.0.146107 was used to remove overhang adapter sequences, generate subreads, and produce circular consensus sequences. Circular consensus sequence reads with a mean per-read quality score <20 were discarded to generate HiFi reads. Reads shorter than 1000 bp were removed using Filtlong v0.2.1. Assemblies were generated using Bactopia v3.0, together with previously obtained high-quality Illumina MiSeq short-read data where available, and assembly graphs were inspected using Bandage v0.8.1.

Genome annotation was performed using the DFAST pipeline v1.3.6 (Tanizawa et al., 2018). A representative DFAST web run log was generated on September 23, 2025. Coding sequences were predicted using MetaGeneAnnotator v2008/08/19 with Bacterial Translation. tRNA, rRNA, and CRISPR features were predicted using Aragorn v1.2.38, Barrnap v0.8, and CRT v1.2, respectively. Functional annotation was performed by DBsearch against DFAST-default.ref using GHOSTX v1.3.6, with e-value, query-coverage, and subject-coverage cutoffs of 1 × 10^−6^, 75%, and 75%, respectively. The DFAST-default reference database contained 381,721 sequences (database version 1.2.1; modified February 14, 2024). Possible pseudogenes were screened using LAST v959 and BLASTP v2.6.0. DFAST output files were organized by sample for downstream comparative analyses. Genome statistics, including total sequence length, number of sequences, N50, G+C content, final coding sequences, rRNAs, tRNAs, CRISPRs, and pseudogene candidates, were summarized from DFAST output tables.

The annotated assemblies used for comparative analyses had the following sizes and G+C contents: NN19, 3060,055 bp and 61.9% G+C; KJ09, 3054,235 bp and 62.0% G+C; OP1, 3054,081 bp and 62.0% G+C; IROP2, 2858,869 bp and 62.2% G+C; and NTG18, 2955,333 bp and 62.0% G+C. DFAST final output tables contained 2904 CDSs for NN19, 2903 for KJ09, 2898 for OP1, 2721 for IROP2, and 2823 for NTG18.

Raw genome-sequencing and RNA-seq read data were deposited under DDBJ BioProject accession PRJDB37616; detailed Run and BioSample accessions are provided in the Data Availability section.

### 5.10 Genome-based taxonomy using TYGS

Whole-genome taxonomic analyses of NN19, KJ09, OP1, and IROP2 were performed using the TYGS (https://tygs.dsmz.de) (Meier-Kolthoff and Göker, 2019). Genome sequences were uploaded to the TYGS, and the analysis results were returned on April 30, 2026. Closely related type strains were determined by MASH comparison against TYGS type-strain genomes (Ondov et al., 2016) and by 16S rRNA gene sequence matching. Pairwise genome comparisons were performed using the Genome BLAST Distance Phylogeny (GBDP) approach, and dDDH values and confidence intervals were calculated using GGDC 4.0 with the recommended settings (Meier-Kolthoff et al., 2013). The recommended formula d4 was used for species-level interpretation. Phylogenomic trees were inferred from GBDP distances using FastME 2.1.6.1 with SPR postprocessing and 100 pseudo-bootstrap replicates (Lefort et al., 2015), midpoint-rooted, and visualized using PhyD3 (Kreft et al., 2017). Type-based species clustering data were interpreted using the 70% dDDH species boundary, and subspecies clustering data were interpreted using the 79% dDDH threshold.

### 5.11 Pairwise genome alignment and definition of the NTG18 deletion-derived GAP2 region

Pairwise genome alignments among CPT-transforming isolates and NTG18 were performed using the MUMmer4 dnadiff workflow (MUMmer v4.0.1; dnadiff v1.3) (Marçais et al., 2018). Genome FASTA headers were standardized prior to comparison. For each pairwise comparison, dnadiff was run with the renamed reference and query FASTA files using the ‘-p’ option to set the output prefix. The dnadiff workflow internally ran ‘nucmer –maxmatch’, followed by ‘delta-filter −1′ and ‘delta-filter -m’, ‘show-coords -rclTH’, ‘show-snps -rlTHC’, and ‘show-diff -rH/-qH’. dnadiff reports were used to summarize aligned bases and average nucleotide identity-like identity values for each pairwise comparison. To identify regions present in NN19 but absent from NTG18, NN19 was used as the reference genome and NTG18 as the query genome. Reference-specific regions were extracted from ‘dnadiff show-diff -rH’ output and intersected with DFAST gene annotations to identify genes overlapping NN19-specific regions.

The pNN19–1 interval encompassing the major NN19 regions absent from NTG18 was designated GAP2. GAP2 was mapped to NN19 using NUCmer alignment and corresponded to a 97,770-bp region of NN19 pNN19–1 from positions 401,171 to 498,940 with 100% nucleotide identity. This interval included the major R2 and R3 regions absent from NTG18. Genes overlapping the deleted regions were then classified using DFAST product annotation and manually grouped into broad functional categories that included oxygenases, oxidoreductases, hydrolases, transporters, transcriptional regulators, and hypothetical proteins.

### 5.12 HiFi-read validation of NTG18 structural differences and SNVs

Assembly-derived SNV candidates from the one-to-one NN19-NTG18 dnadiff alignments were validated against the raw NTG18 HiFi reads. Variant-centered reference and alternate haplotypes of 31 to 1001 bp were searched directly against these reads, and a candidate was retained when the locus-specific alternate haplotype was supported by at least five HiFi reads. For each assembly-defined NN19-specific region and retained control region, 20 unique 31-bp probes were searched against the NTG18 HiFi reads. R2, R3, and pNN19–2 had no supporting reads, whereas retained control regions showed robust support. SNVs were compared with the corresponding KJ09 and OP1 alleles to distinguish NTG18-specific changes from variants shared with CPT-transforming strains. A summary of the validation criteria and same-lineage comparison workflow is provided in Supplementary Table S4E.

### 5.13 Identification and prioritization of GAP2 candidate genes

GAP2 genes were evaluated using comparative-genomic, functional-annotation, cross-lineage conservation, and expression evidence. DFAST product annotations were screened for functions plausibly related to aromatic or alkaloid transformation, and gene presence in CPT-transforming strains was compared with absence from the non-transforming mutant NTG18. LOCUS_040, LOCUS_070, LOCUS_080, and LOCUS_090 were designated the core candidate module because their predicted functions were consistent with oxygenation or ring-cleavage reactions, homologs were conserved in the distinct IROP2 lineage, and the genes were expressed during growth of NN19 on CPT-containing media. All remaining genes were classified as other GAP2 genes and interpreted as representing a broader accessory-region context rather than ranked candidates.

### 5.14 Accessory-genome functional classification

The broader genomic context of the GAP2 candidate region was evaluated by classifying coding sequences in NN19 regions absent from NTG18 and in the GAP2 interval into broad functional categories using DFAST product annotations. Categories included oxidation-reduction, hydrolase-related, transport or receptor, transcriptional regulator, hypothetical protein, and other functions. For whole-genome contextual summaries, NN19 coding sequences were additionally classified by keyword-based parsing of product descriptions from the RNA-seq expression table. This classification was used only for descriptive accessory-genome analysis and not to assign specific enzymatic reactions.

RNA-seq expression values for the accessory-genome summaries were taken from the HISAT2-featureCounts TPM table generated from three NN19 CPT-growth replicate libraries. Because no matched non-CPT RNA-seq control was available, expression summaries were interpreted as activity during growth on CPT-containing media rather than CPT induction.

### 5.15 Homology searching of GAP2 candidates in IROP2

To determine whether IROP2 carries homologs of selected NN19 GAP2 genes, protein sequences of 12 functionally selected candidates, including the four core candidate–module proteins, were searched against the IROP2 DFAST-predicted protein set using BLASTp from BLAST+ v2.17.0 (Camacho et al., 2009). A local protein database was generated from the IROP2 DFAST protein FASTA file using makeblastdb, and BLASTp was run with an e-value cutoff of 1 × 10^−3^. Tabular output was generated using outfmt 6 with the fields ‘qseqid sseqid pident length qlen slen qstart qend sstart send evalue bitscore qcovs’. Best hits were summarized based on amino acid identity, query coverage, and product annotation. Hits with high identity and near-complete query coverage were considered strong homologs; lower-identity full-length hits were considered moderate homologs. The genomic positions of IROP2 best-hit genes were inspected using DFAST feature tables to determine whether homologs were clustered on pIROP2.

### 5.16 RNA-seq data and expression screening of NN19 during growth on CPT-containing media

RNA-seq data were generated from NN19 grown on media containing CPT as the sole added carbon source. LB precultures of NN19 were inoculated at 1% (v/v) into six 100-mL inorganic salts medium cultures containing 10 mg of CPT, and the cells were harvested after 48 h. Total RNA was extracted from each sample using ISOGEN (Nippon Gene) according to the manufacturer’s protocol and dissolved in diethyl pyrocarbonate-treated water. The RNA concentration was determined using a Qubit RNA BR assay kit and Qubit 2.0 fluorometer (Thermo Fisher Scientific). Three samples exhibiting high RNA quantity and quality were selected for library preparation.

To control RNA quality before library preparation, total RNA was quantified using a Quantus Fluorometer and QuantiFluor RNA system (Promega), and RNA integrity was assessed using a 5200 Fragment Analyzer System with an Agilent HS RNA kit (Agilent Technologies). Genomic DNA was removed using RNA Clean & Concentrator-5 with DNase I (Zymo Research, Irvine, CA, USA). Ribosomal RNA was depleted from total RNA using a Ribo-Zero Plus rRNA Depletion kit (Illumina, San Diego, CA, USA). Strand-specific RNA-seq libraries were prepared using an NEBNext Ultra II Directional RNA Library Prep kit (New England Biolabs, Ipswich, MA, USA). In brief, first-strand cDNA was synthesized by reverse transcription using rRNA-depleted RNA as the template, and second-strand cDNA was synthesized using incorporation of dUTP instead of dTTP to preserve strand information. After end repair, phosphorylation, 3′-dA tailing, and adaptor ligation, dUTP-containing strands were selectively cleaved using USER enzyme (New England Biolabs), yielding strand-specific libraries. Sequencing was performed on an Illumina NovaSeq X Plus instrument (Illumina) in PE150 mode (150 bp x 2 paired-end). Three paired-end replicate libraries were analyzed: NN19_CPT_rep3, NN19_CPT_rep4, and NN19_CPT_rep5. Because matched CPT-free or alternative-carbon-source libraries were not included, the RNA-seq data were used to prioritize genes transcribed during growth with CPT and not to assess CPT-specific induction.

Paired-end reads were mapped to the NN19 genome assembly using HISAT2 v2.2.2 with the ‘–no-spliced-alignment –no-unal’ options. Alignments were sorted and indexed using samtools v1.23.1. (Li et al., 2009) CDS-level fragment counts were obtained using featureCounts v2.1.1 in paired-end, unstranded mode with an SAF annotation generated from DFAST-predicted CDS records. Counts were normalized to TPM using CDS lengths, and expression ranks were calculated across all NN19 CDSs based on mean TPM across the three CPT-growth replicates. This RNA-seq analysis was used as an expression screen under CPT-growth conditions but not used for differential induction analysis because no non-CPT control libraries were available.

### 5.17 Figure generation and data visualization

Genome maps and GAP2/pIROP2 homolog maps were generated from DFAST feature tables and comparative-genomic outputs using custom Python scripts with Matplotlib v3.10.8 (Hunter, 2007) and exported as vector graphics where possible (Fig. 2).

## Funding

This research did not receive any specific grant from funding agencies in the public, commercial, or not-for-profit sectors.

## CRediT authorship contribution statement

**Hisashi Takeda:** Conceptualization, Methodology, Investigation, Data curation, Formal analysis, Visualization, Writing – original draft. **Nagomi Takahashi:** Investigation, Data curation. **Kyle Shuta Ikeda Kakiura:** Investigation, Data curation. **Hitomi Suzuki:** Investigation, Data curation. **Tatsuki Kijinami:** Investigation, Data curation. **Masako Horiuchi:** Formal analysis, Validation. **Daigo Wakana:** Formal analysis, Validation, Data curation. **Hiroshi Sudoh:** Conceptualization, Methodology, Validation. **Tomoo Hosoe:** Supervision, Project administration, Writing – review & editing.

## Declaration of competing interest

The authors declare that they have no known competing financial interests or personal relationships that could have appeared to influence the work reported in this paper.

## Data Availability

Raw genome-sequencing and RNA-seq read data generated in this study are available under DDBJ BioProject accession PRJDB37616 and DRA Run accessions DRR1063135-DRR1063142. BioSample accession numbers include SAMD01915359 for KJ09, SAMD01915360 for NN19, SAMD01915361 for OP1, SAMD01915362 for IROP2, and SAMD01915363 for NTG18. Run accession numbers include DRR1063135-DRR1063136 for KJ09, DRR1063137-DRR1063138 and DRR1063142 for NN19, DRR1063139 for OP1, DRR1063140 for IROP2, and DRR1063141 for NTG18.
