# Supplementary material for: Camptothecin transformation in plant-associated Sphingobium lineages is linked to a plasmid accessory region
Source: Curr Res Microb Sci. 2026 Oct 23;11:100677. doi: 10.1016/j.crmicr.2026.100677 (PMC13744829; doi:10.1016/j.crmicr.2026.100677)
Supplement: Supplementary file 1 [file mmc1.pdf]

Camptothecin transformation in Plant-associated *Sphingobium* lineages is linked to a plasmid accessory region

Hisashi Takeda, Nagomi Takahashi, Kyle shuta Ikeda Kakiura, Hitomi Suzuki, Tatsuki Kijinami, Masako Horiuchi, Daigo Wakana, Hiroshi Sudoh, Tomoo Hosoe

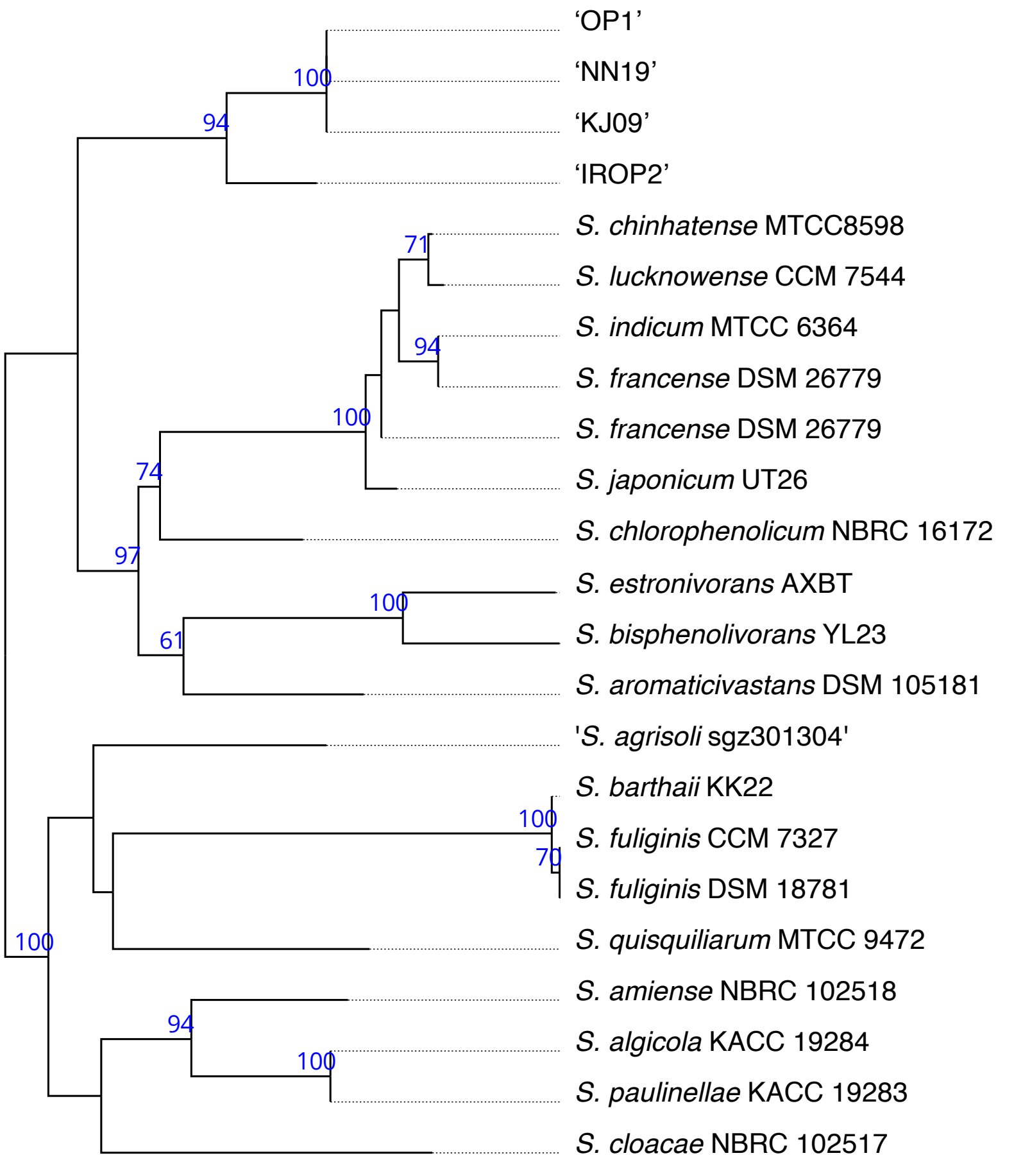

Supplementary Figure S1. 16S rRNA gene phylogenetic tree of the CPT-transforming isolates and related Sphingobium strains. The tree shows OP1, NN19, KJ09, IROP2, and related *Sphingobium* strains. Numbers at nodes indicate bootstrap support values. This analysis provides initial genus-level placement and complements the genome-scale Type (Strain) Genome Server analysis shown in Figure 2.

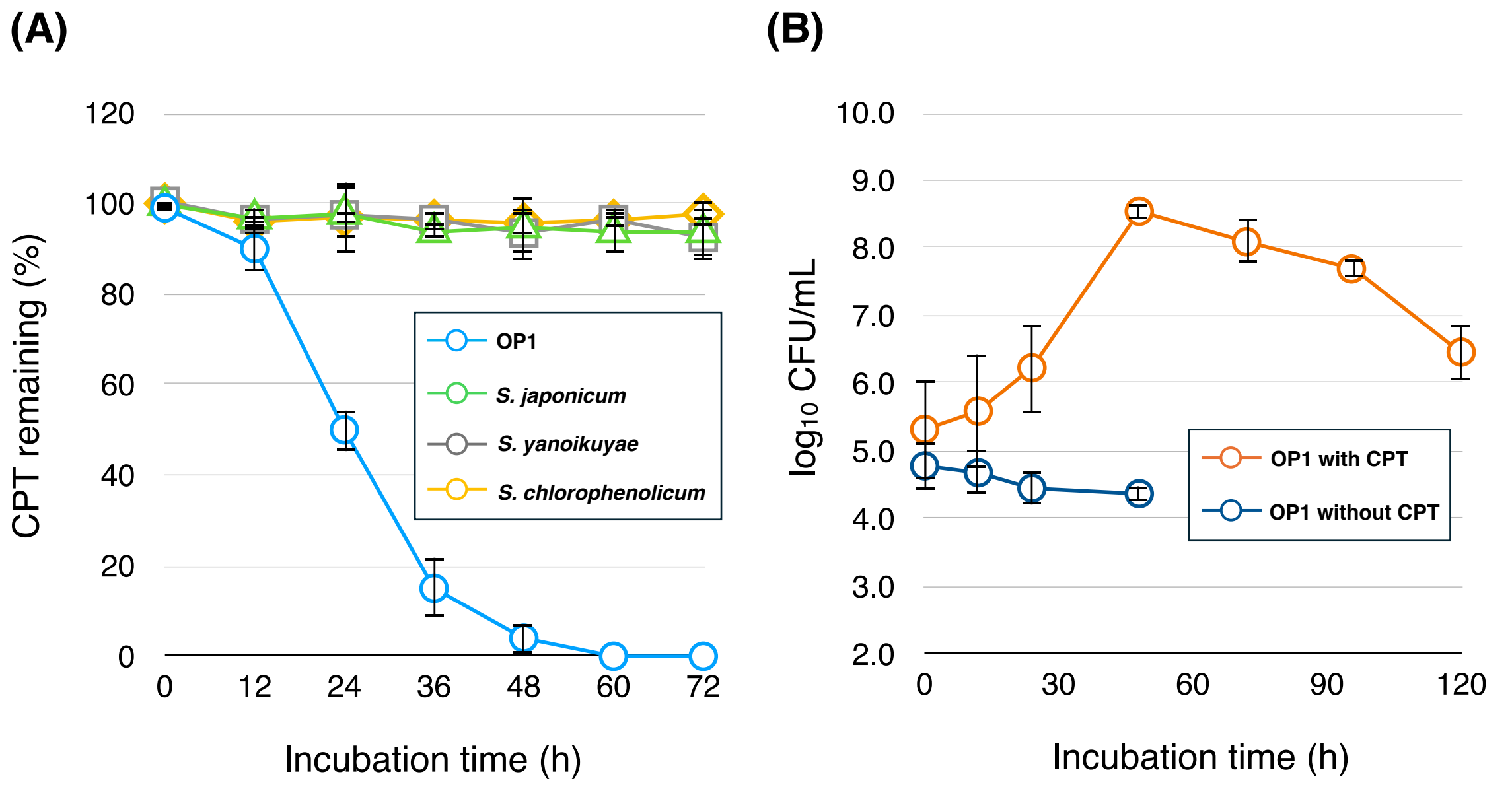

Supplementary Figure S2. Camptothecin depletion and growth of strain OP1 in inorganic salts medium. (A) Camptothecin (CPT) remaining during incubation with strain OP1 or the comparator strains *Sphingobium japonicum* UT26, *S. chlorophenolicum* ATCC 33790, and *S. yanoikuyae* ATCC 51230 in CPT-supplemented WM inorganic salts medium. Values are expressed relative to the amount detected at 0 h. Symbols and error bars represent the mean ± standard deviation (SD) of three independent cultures (n = 3). CPT was below the limit of quantification in the OP1 cultures at 60 and 72 h. (B) Viable counts of OP1 cultured in WM inorganic salts medium with or without CPT as the sole added carbon source. Symbols and error bars represent the mean ± SD of log10-transformed colony-forming units (CFU) per milliliter from three independent cultures (n = 3). The mean for the CPT-free condition is shown through 48 h; one culture was below the detection limit (<100 CFU/mL) at 72 h, and all three were below the detection limit at 96 and 120 h.

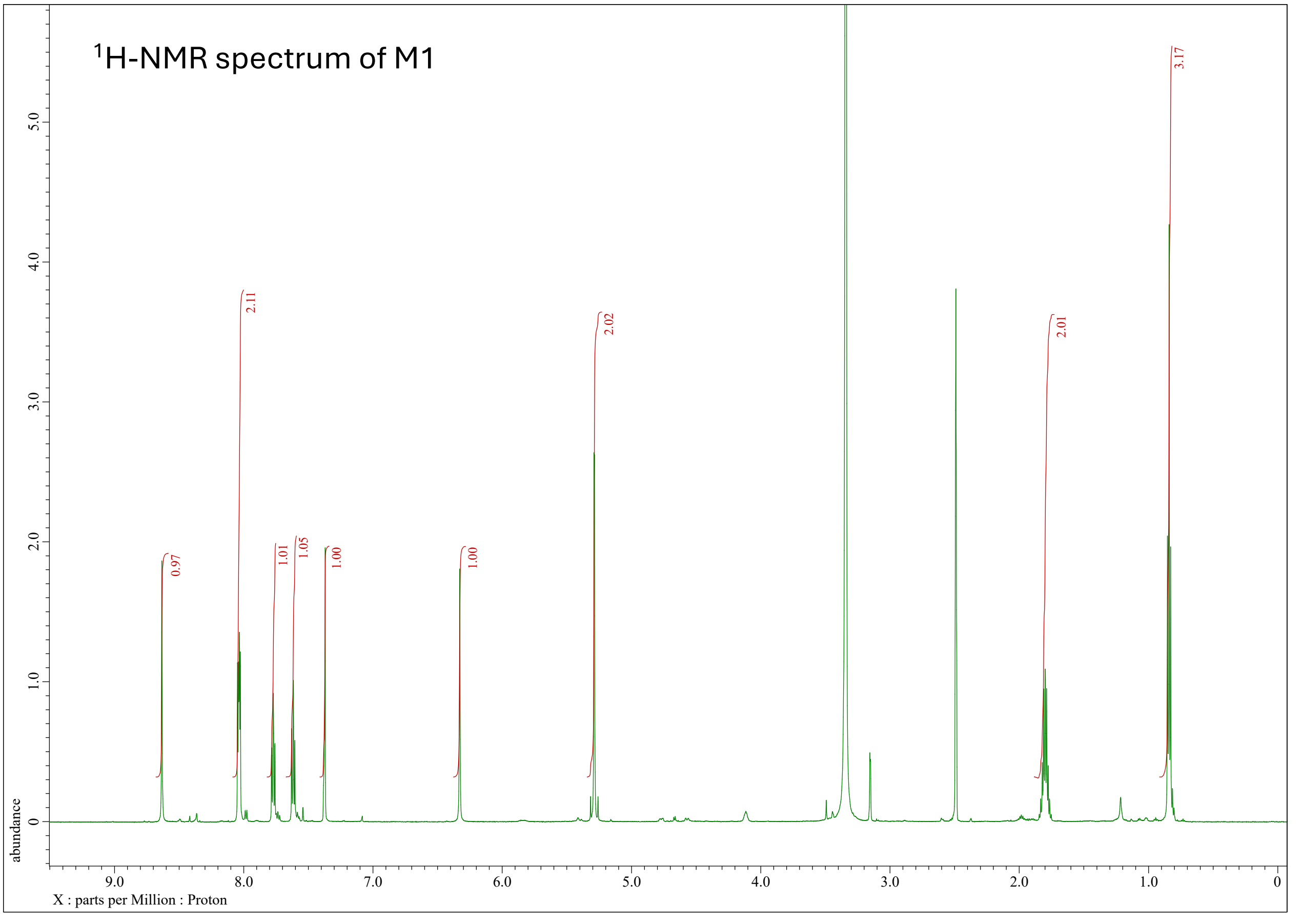

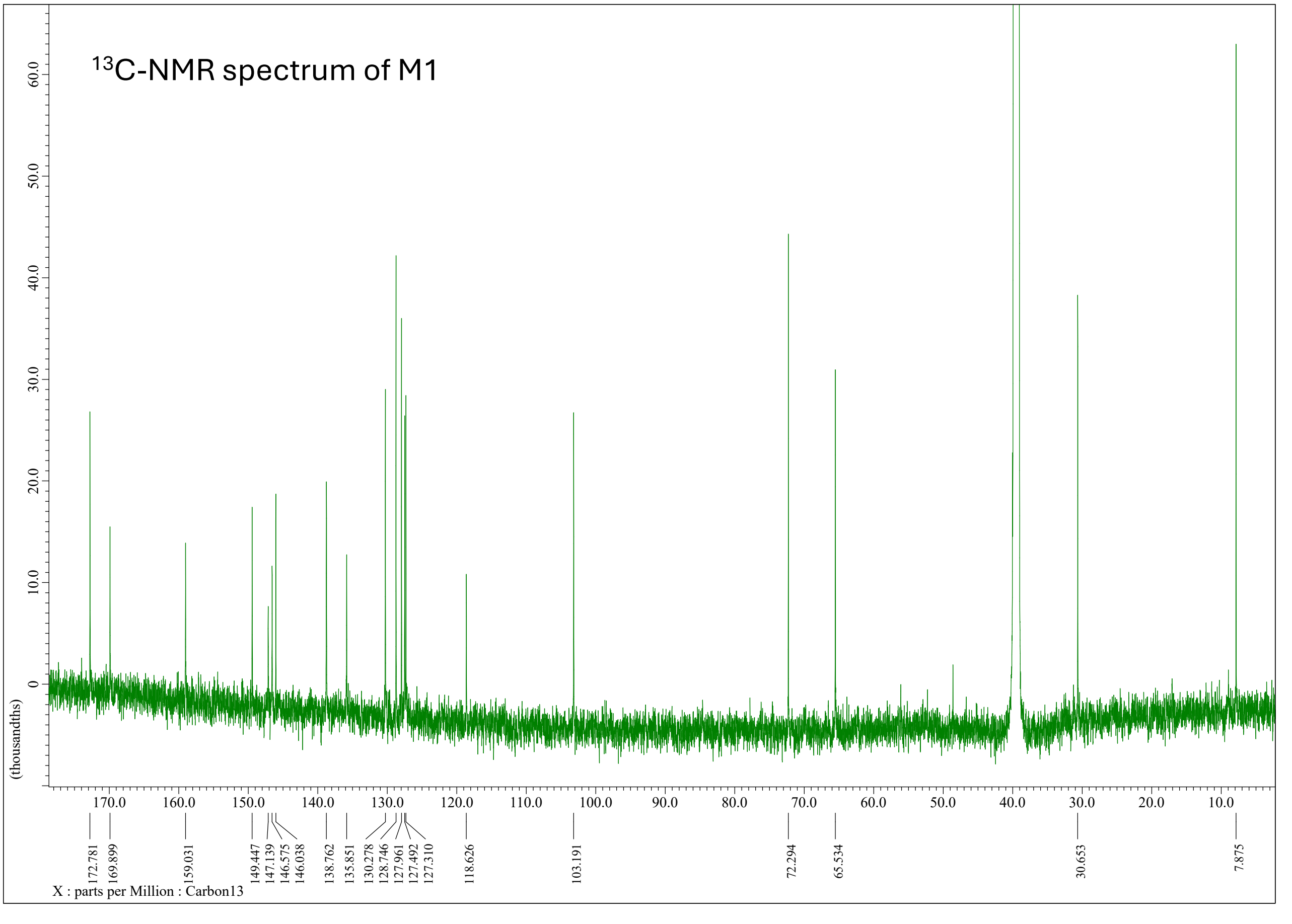

Supplementary Figure S3. One-dimensional nuclear magnetic resonance spectra of M1. The upper panel shows the proton nuclear magnetic resonance (^{1}H-NMR) spectrum of purified M1, and the lower panel shows the carbon-13 nuclear magnetic resonance (^{13}C-NMR) spectrum of purified M1.

Supplementary Figure S3

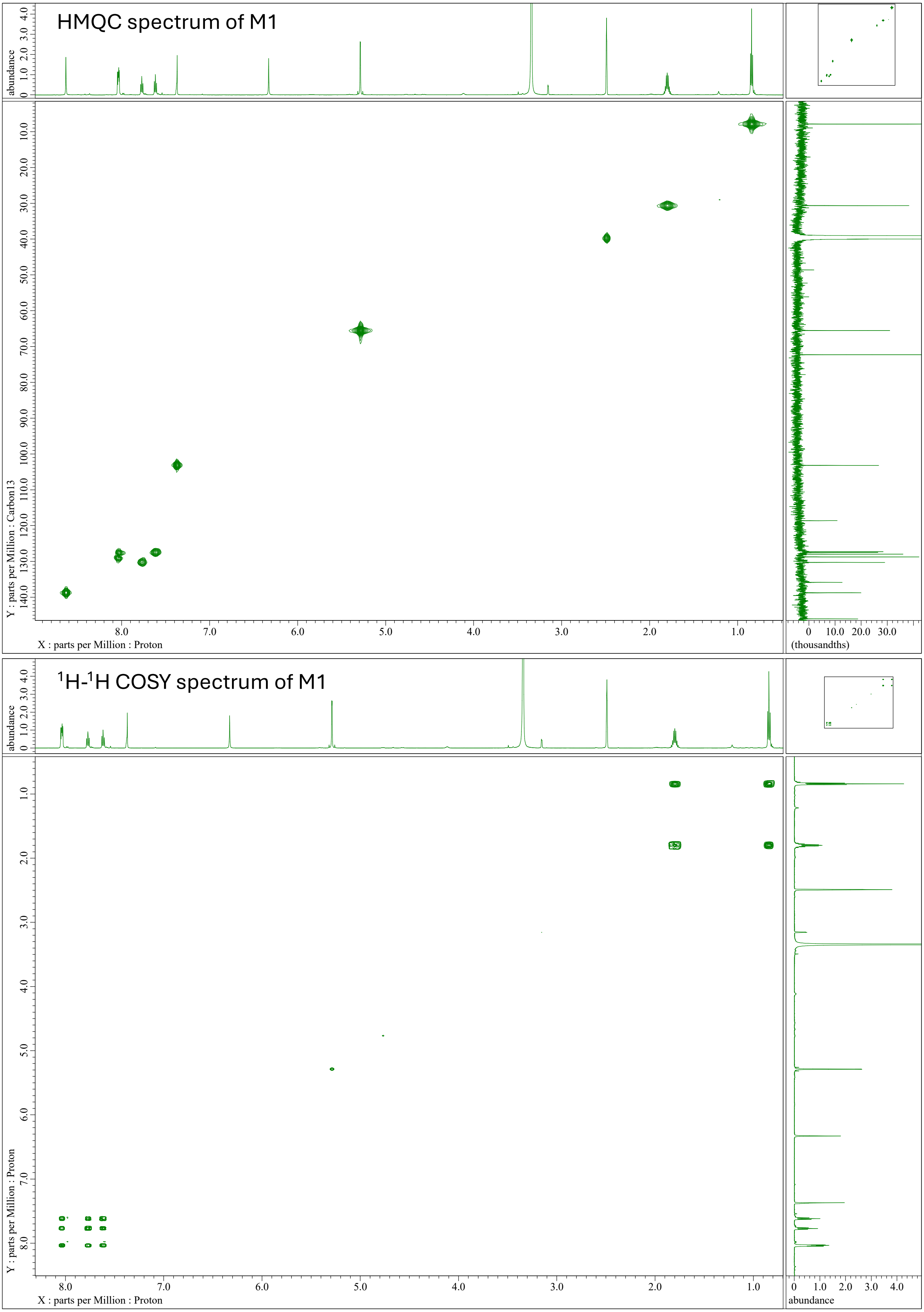

Supplementary Figure S4. Two-dimensional nuclear magnetic resonance spectra of M1. The upper panel shows the heteronuclear multiple quantum coherence (HMQC) spectrum of purified M1, and the lower panel shows the proton-proton correlation spectroscopy (^{1}H-^{1}H COSY) spectrum of purified M1.

Supplementary Figure S4

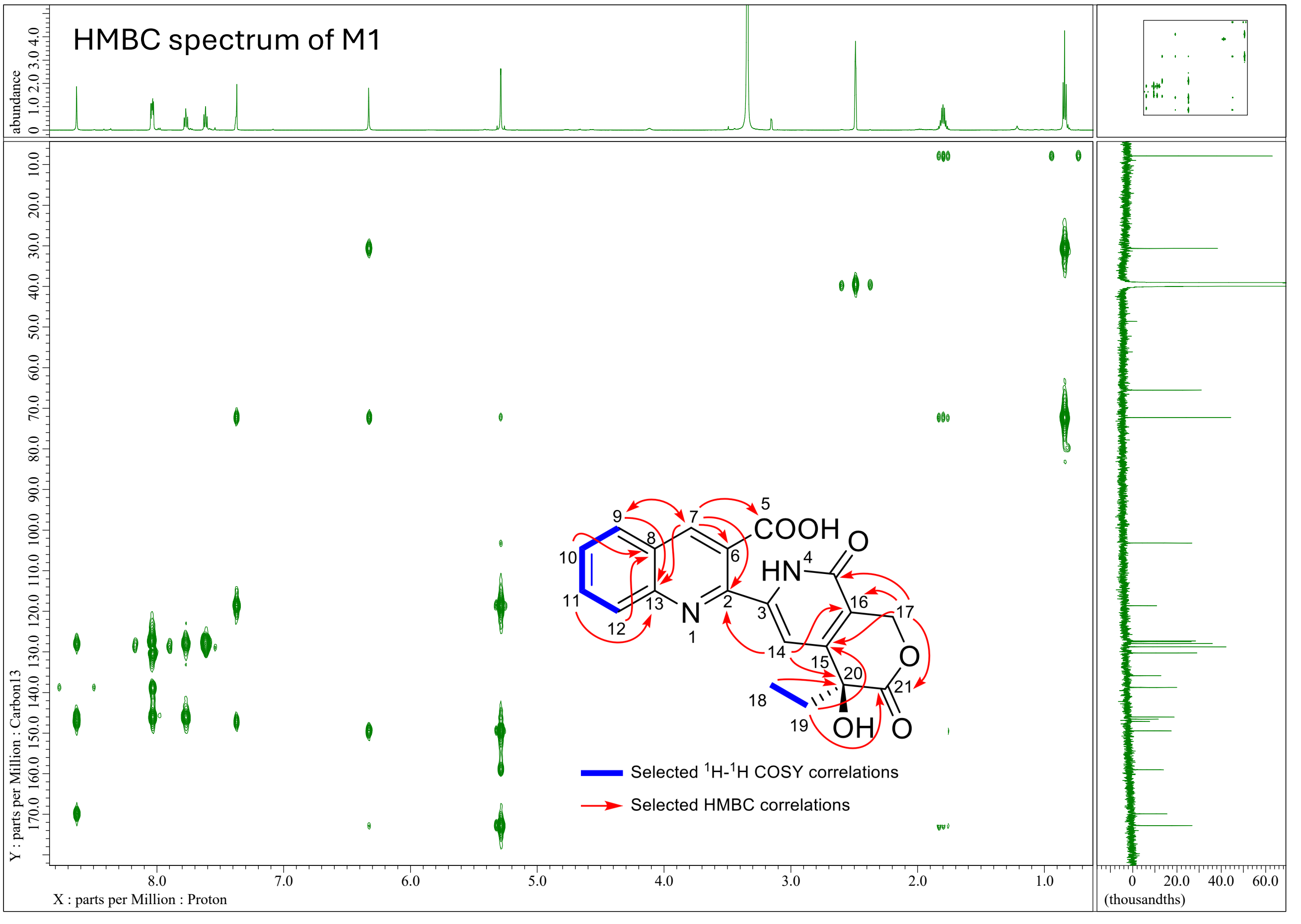

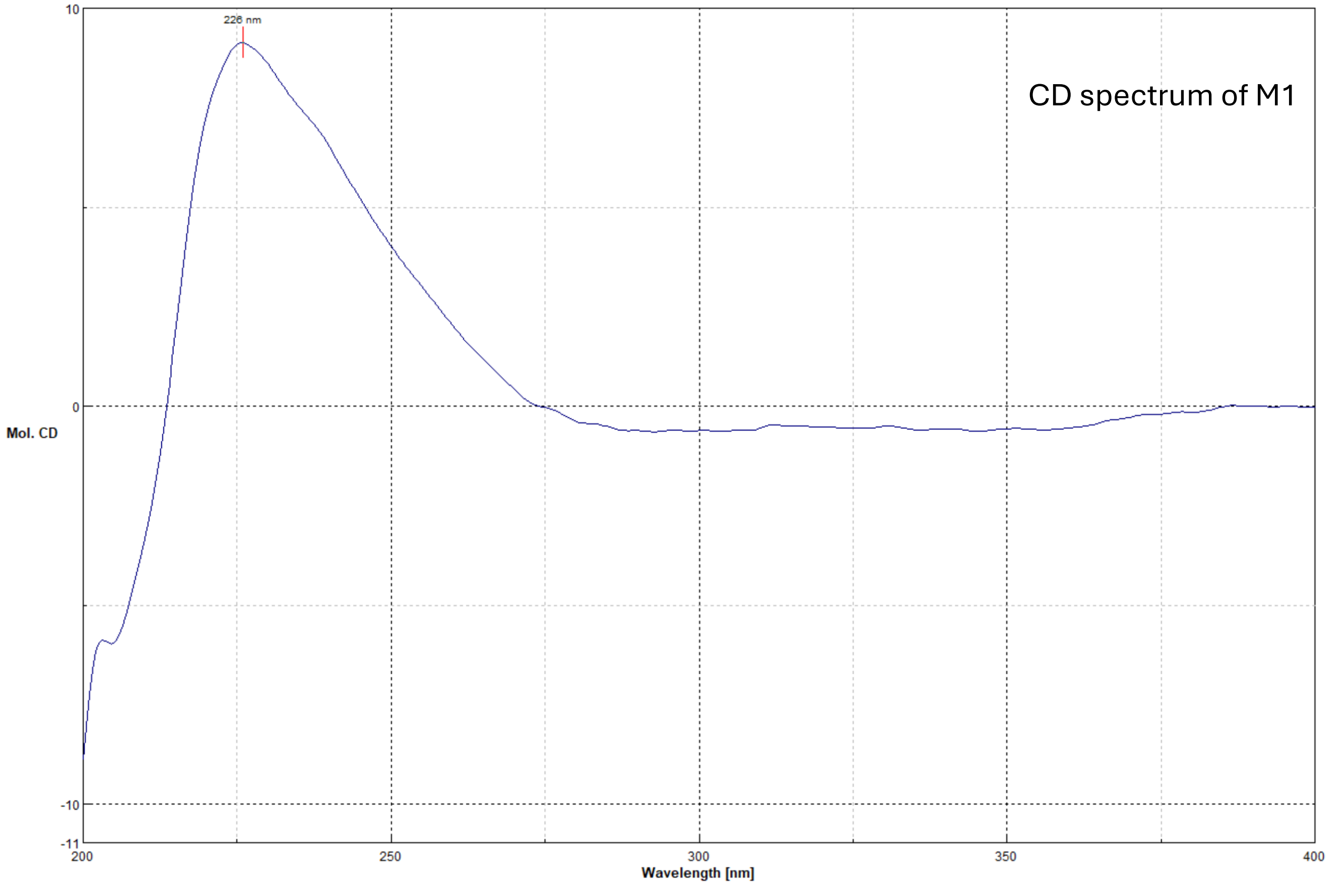

Supplementary Figure S5. Heteronuclear multiple bond correlation and circular dichroism spectra of M1. The upper panel shows the heteronuclear multiple bond correlation (HMBC) spectrum, including selected ^{1}H-^{1}H COSY and HMBC correlations used for M1 structural assignment. The lower panel shows the circular dichroism spectrum of M1.

Supplementary Figure S5

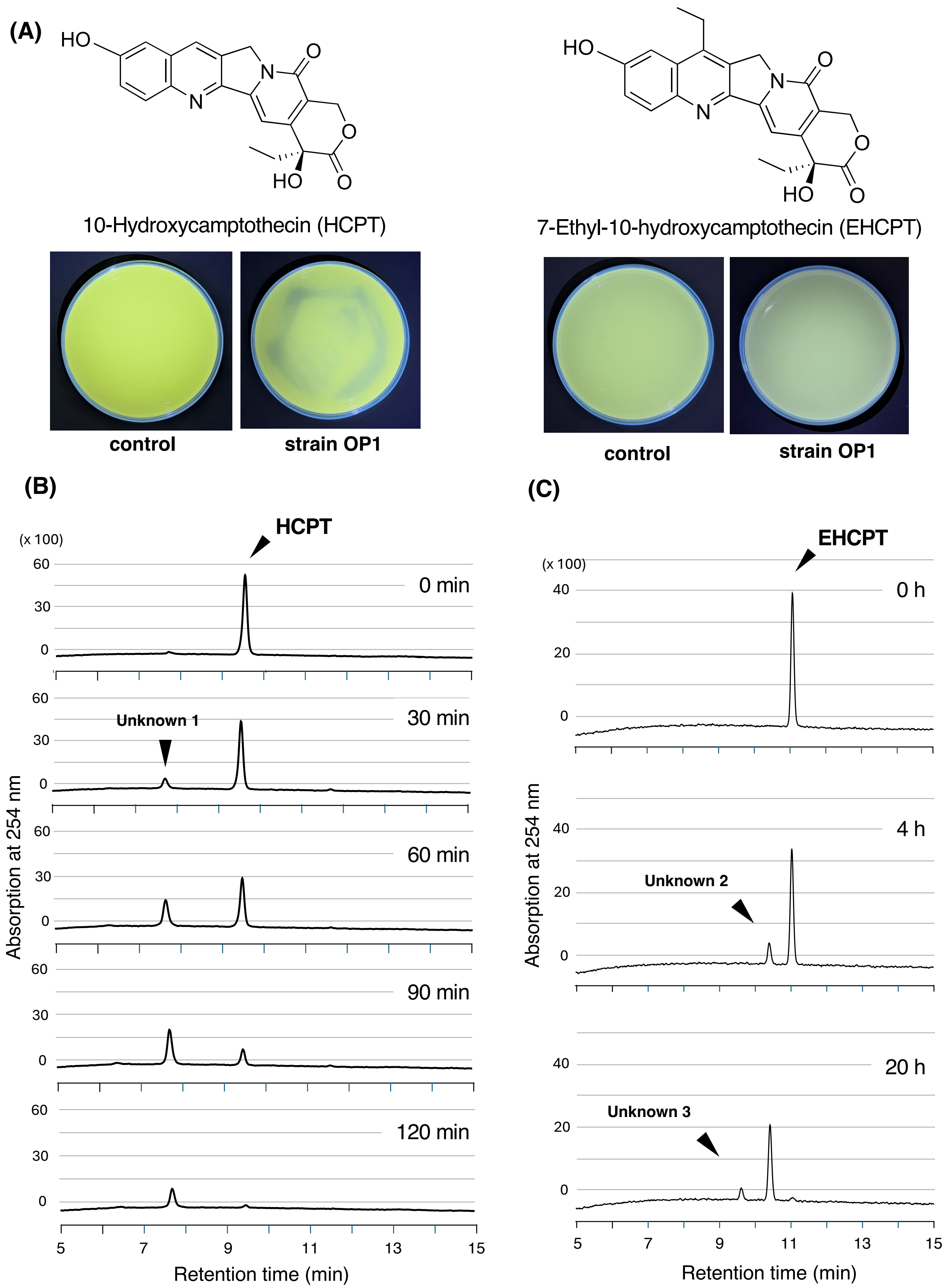

Supplementary Figure S6. Transformation of CPT analogs by OP1. (A) Structures of HCPT and EHCPT with representative agar-plate images. (B, C) Time-course HPLC chromatograms of OP1 resting-cell reactions with HCPT and EHCPT, respectively. Arrowheads indicate substrate and newly detected product peaks.

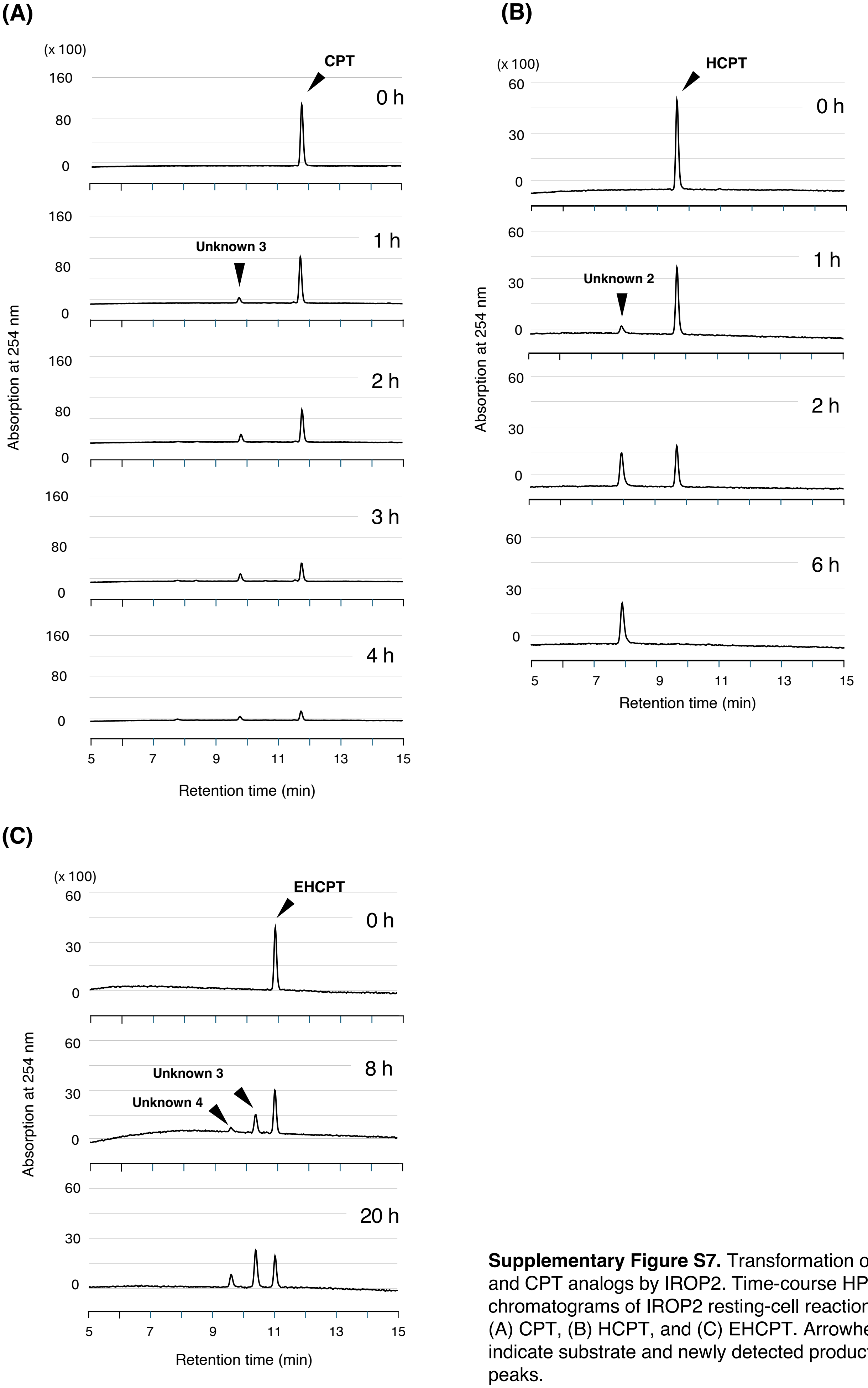

Supplementary Figure S7. Transformation of CPT and CPT analogs by IROP2. Time-course HPLC chromatograms of IROP2 resting-cell reactions with (A) CPT, (B) HCPT, and (C) EHCPT. Arrowheads indicate substrate and newly detected product peaks.

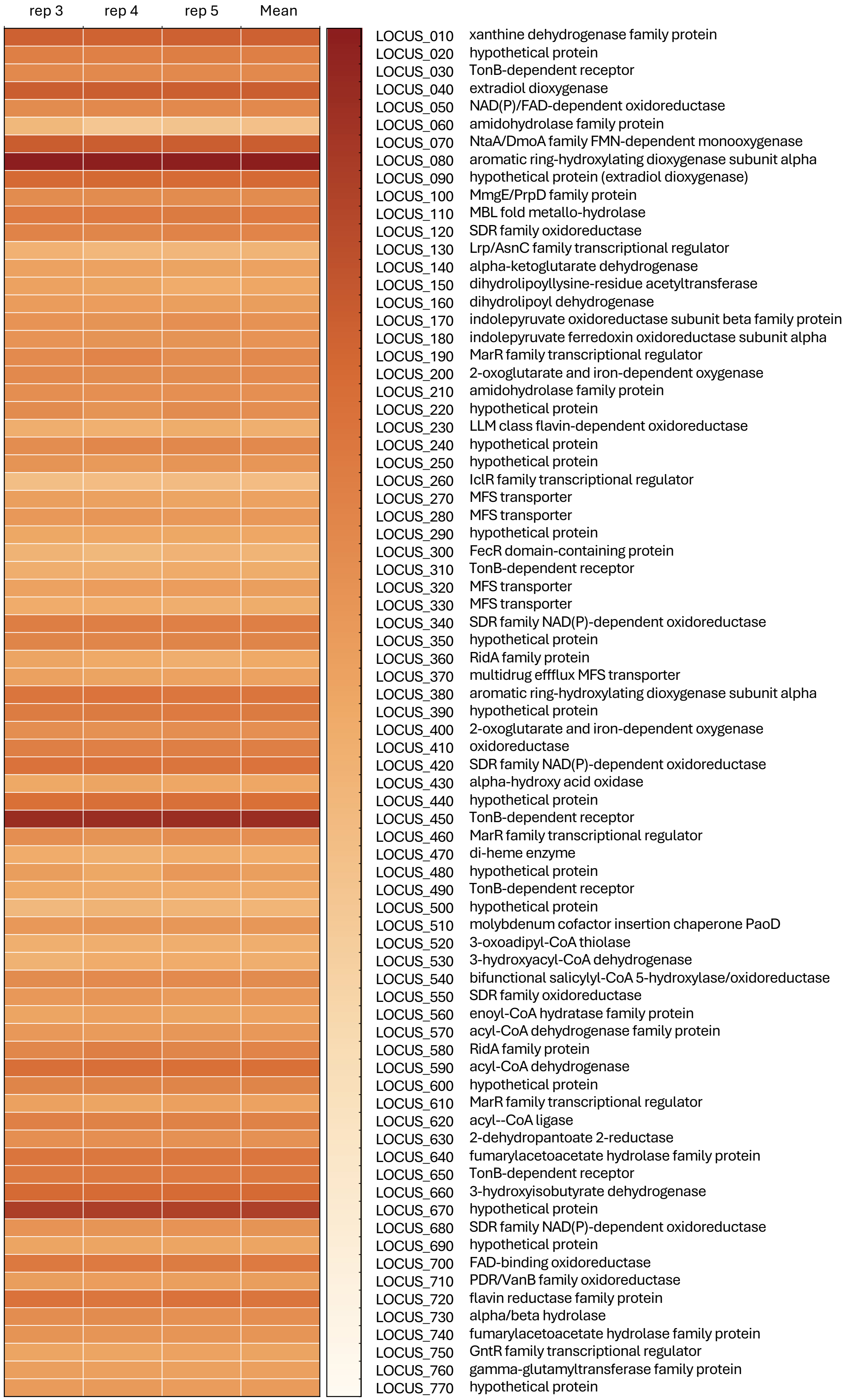

Supplementary Figure S8. Expression heatmap of genes in the GAP2 candidate region during growth with CPT. Heatmap of transcripts per million (TPM) values from HISAT2-featureCounts analysis for GAP2 genes across three NN19 biological replicates grown with CPT as the sole added carbon source. Genes are ordered by genomic position across the deletion-defined GAP2 interval. The data identify genes expressed during growth on CPT but do not establish CPT-induced transcription because a non-CPT RNA-seq control was not included.
